# Statin–Ezetimibe Attenuates Hepatic Inflammation and Fibrosis in a Diet- and Toxin-Induced Steatohepatitis Model, Associated with Macrophage NF-κB Inhibition

**DOI:** 10.3390/cells15181700

**Published:** 2026-09-19

**Authors:** Seul Ki Han, Jin Suk Lee, Su Jung Park, Mi Ra Lee, Yu Jin Yi, Kangchan Choi, Soon Koo Baik, Moon Young Kim

**Affiliations:** 1 Department of Internal Medicine, Yonsei University Wonju College of Medicine, Wonju 26426, Republic of Korea; 2 Regeneration Medicine Research Center, Yonsei University Wonju College of Medicine, Wonju 26426, Republic of Korea; 3 Cell Therapy and Tissue Engineering Center, Yonsei University Wonju College of Medicine, Wonju 26426, Republic of Korea; 4 Mitohormesis Research Center, Yonsei University Wonju College of Medicine, Wonju 26426, Republic of Korea

**Keywords:** metabolic dysfunction-associated steatohepatitis, macrophage activation, NF-κB, hepatic fibrosis, statin, ezetimibe

## Abstract

**Highlights:**

**What is already known?**

- The combination of statin and ezetimibe is a standard lipid-lowering regimen widely co-prescribed in patients with MASH and dyslipidemia; the ESSENTIAL randomized controlled trial showed this combination significantly reduces hepatic fat content compared to statin monotherapy in patients with MASLD.
- Macrophage-driven NF-κB signaling is a central mediator of hepatic inflammation and fibrogenic activation in MASH, yet whether clinically used lipid-lowering agents modulate this pathway in the liver has not been directly demonstrated.

**What is new?**

- This study shows that the statin–ezetimibe combination suppresses NF-κB p65 nuclear translocation in cultured macrophage models, supporting an association between macrophage NF-κB inhibition and the hepatic anti-inflammatory effects observed in vivo, a candidate mechanism contributing to the hepatic anti-inflammatory effects in a multi-hit MASH model.
- The anti-fibrotic benefit appears to be at least partly indirect, likely involving macrophage suppression rather than direct hepatic stellate cell inhibition, thereby clarifying the probable mechanistic hierarchy of this combination.
- These findings provide pre-clinical mechanistic support for the hepatoprotective potential of a clinically established and cost-effective drug combination, positioning it as a viable drug-repurposing candidate for prospective MASH trials.

**Abstract:**

**Background and Aims:** Metabolic dysfunction-associated steatotic liver disease (MASLD) affects over 30% of adults globally; its progressive form, metabolic dysfunction-associated steatohepatitis (MASH), has limited pharmacological options beyond the recently approved resmetirom. The statin–ezetimibe combination, widely co-prescribed for dyslipidemia in patients with MASH, significantly reduces hepatic fat versus statin monotherapy in the ESSENTIAL randomized trial, yet macrophage-driven mechanisms linking this lipid-lowering combination to hepatic inflammation and fibrosis remain poorly characterized. We investigated these mechanisms using in vivo and in vitro approaches. **Methods:** Male C57BL/6 mice (*n* = 6/group; 6 groups) received a high-fat diet (HFD; 45% kcal) plus thioacetamide (TAA; 300 mg/kg twice-weekly, 8 weeks) to establish MASH with advanced fibrosis. Simvastatin (5 mg/kg) and/or Ezetimibe (10 mg/kg) were added to the diet for 4 weeks. Blinded hepatic histology (H&E, Picrosirius red, Oil Red-O), qPCR, immunohistochemistry, and Western blot were performed. Macrophage polarization and NF-κB nuclear translocation were assessed in RAW 264.7 cells and thioglycolate-elicited peritoneal macrophages by flow cytometry and immunofluorescence. **Results:** Combination therapy significantly reduced steatosis, inflammation, and fibrosis versus HFD-TAA controls. Pro-inflammatory (IL-1β, IL-6, TNF-α, iNOS) and fibrosis-related (collagen-I, α-SMA, TGF-β1) markers were markedly suppressed (*p* < 0.01). In vitro, the combination reduced the proportion of iNOS^+^ macrophages to approximately 50% of the LPS control (normalized to each experiment), although statistical significance was not reached; monotherapies showed weaker and variable trends and inhibited NF-κB p65 nuclear translocation in cultured macrophages (Holm-adjusted *p* < 0.01); MAPK signaling was not significantly altered under the present conditions. Anti-fibrotic effects were consistent with a macrophage-mediated mechanism, with no direct stellate cell suppression observed by LX-2 assays. **Conclusions**: Statin–Ezetimibe combination exerts anti-inflammatory and anti-fibrotic effects in a multi-hit murine model of MASH with advanced fibrosis associated with macrophage NF-κB inhibition in vitro and with reduced hepatic inflammatory signaling in vivo. These findings provide mechanistic rationale for the hepatic benefits of this widely used regimen in MASH patients with dyslipidemia, supporting its evaluation as a drug-repurposing strategy.

## 1. Introduction

Metabolic dysfunction-associated steatotic liver disease (MASLD) has emerged as a leading cause of chronic liver disease worldwide, affecting over 30% of adults globally, and can progress to metabolic dysfunction-associated steatohepatitis (MASH), advanced hepatic fibrosis, cirrhosis, and hepatocellular carcinoma [1,2]. The pathogenesis is primarily driven by hepatic lipid accumulation, which precipitates insulin resistance and inflammation [3,4]. Accumulated lipids directly contribute to the generation of reactive oxygen species (ROS) and pro-inflammatory cytokines [5,6]; consequently, multiple factors, including mitochondrial dysfunction, endoplasmic reticulum stress, and oxidative stress, play pivotal roles in the progression from simple steatosis to steatohepatitis [7,8]. While dietary modifications and exercise therapy remain the foundational strategies for managing MASLD, numerous pharmacological agents intended to replace or complement these approaches are still undergoing clinical evaluation [9,10].

Statins, 3-hydroxy-3-methylglutaryl coenzyme A (HMG-CoA) reductase inhibitors, are widely utilized as first-line therapy for lowering low-density lipoprotein (LDL) cholesterol [11]. Beyond their lipid-lowering effects, they exhibit pleiotropic properties, including enhancement of endothelial function, increased nitric oxide bioavailability, immunomodulatory activity, and antioxidant and anti-inflammatory effects [12]. Ezetimibe is a complementary lipid-lowering agent that inhibits cholesterol absorption at the intestinal brush border via Niemann-Pick C1-Like 1 (NPC1L1) receptors. When combined with statins, it further reduces LDL cholesterol by decreasing intestinal cholesterol absorption [13]. Clinically, the cardiovascular protective effects of this combination are well established [13], making it a widely recommended therapeutic regimen [14].

Although the anti-inflammatory effects of statins have been well documented [15], their specific role in the treatment of MASLD, particularly in the context of MASH, remains under-investigated. Certain statins, such as simvastatin, have demonstrated anti-fibrotic effects in several experimental models [16,17]; however, ezetimibe alone and the statin–ezetimibe combination have not been thoroughly studied for their potential effects on macrophage-mediated hepatic inflammation and fibrosis. Previous studies have demonstrated that low-dose statin–ezetimibe therapy improves systemic metabolic parameters, including postprandial lipemia and endothelial function, comparably to high-dose statin monotherapy [18,19]; however, its direct impact on hepatic pathological changes remains poorly defined.

Notably, the ESSENTIAL randomized controlled trial demonstrated that ezetimibe (10 mg/day) combined with rosuvastatin (5 mg/day) significantly reduced hepatic fat content by MRI-PDFF versus rosuvastatin monotherapy (mean difference: −3.2%; *p* = 0.020) [20]. The greatest benefit was observed in patients with higher insulin resistance and more advanced hepatic fibrosis, suggesting a hepatoprotective mechanism beyond simple systemic lipid reduction. Concurrently, the 2024 FDA approval of resmetirom as the first pharmacological agent specifically indicated for MASH [21,22] has validated hepatic lipid metabolism and necroinflammation as tractable therapeutic targets; however, limited accessibility and cost considerations underscore the clinical value of repurposing existing, clinically established agents such as the statin–ezetimibe combination.

Prior work employed a simple high-fat diet (HFD)-only model in which fibrosis was modest [23], thereby limiting anti-fibrotic signal detectability. In contrast, our HFD-TAA model reproducibly induces advanced fibrosis closely resembling human MASH histology, providing a more sensitive and clinically relevant assessment platform. Furthermore, NPC1L1-independent anti-inflammatory effects of ezetimibe have been demonstrated in vitro and in models of atherosclerosis [24], providing a mechanistic rationale for its inclusion despite the low hepatic NPC1L1 expression typical of rodents. We therefore hypothesized that statin–ezetimibe combination therapy may attenuate macrophage-mediated hepatic inflammation and fibrosis in MASH, and designed this study to test this hypothesis using both in vivo and in vitro approaches.

## 2. Materials and Methods

### 2.1. Mouse Models with MASH-Related Hepatic Fibrosis

All animal procedures were approved by the Institutional Animal Care and Use Committee of Yonsei University Wonju College of Medicine. Mice were maintained in individually ventilated cages at 22 °C with a 12-h light/12-h dark cycle and provided with food and water ad libitum. Male wild-type mice (C57BL/6, 7 weeks old, 20–24 g) were fed either a standard chow diet or a high-fat diet providing 45% kcal from fat (D12451; Research Diets, New Brunswick, NJ, USA; fat sources: lard 177.5 g/kg and soybean oil 25 g/kg) for 10 weeks. After 2 weeks of HFD feeding, thioacetamide (TAA, 300 mg/kg) was administered via intraperitoneal injection twice weekly for 8 weeks (from week 2 to week 10) to induce hepatic inflammation and fibrosis, while HFD feeding was continued. Four weeks after TAA initiation (i.e., at week 6 of the total experimental period), mice in the treatment groups began pharmacologic therapy with simvastatin (5 mg/kg), ezetimibe (10 mg/kg), or a combination of both, which were mixed into the diet and administered for the final 4 weeks of the study (from week 6 to week 10; Figures S1 and S2). Doses were selected from published rodent steatohepatitis studies [25,26]. During the treatment period, both HFD feeding and TAA administration continued to distinguish pharmacological anti-fibrotic effects from spontaneous regression that occurs upon TAA withdrawal. To verify drug delivery, feed consumption per cage was monitored weekly, and estimated individual drug intake was calculated based on mean body weight and average feed intake.

Thirty-six mice were randomly assigned to six groups (*n* = 6 per group): (1) normal diet control (Control), (2) HFD alone (HFD), (3) HFD with TAA (HFD-TAA; serving as the disease model control), (4) HFD-TAA with ezetimibe (HFD-TAA-EZET), (5) HFD-TAA with simvastatin (HFD-TAA-statin), and (6) HFD-TAA with both agents (HFD-TAA-combination). Before euthanasia, mice were anesthetized using isoflurane, and blood samples were collected by cardiac puncture. Liver tissues were promptly snap-frozen in liquid nitrogen and stored at −80 °C for molecular biological analyses. For morphological evaluation, a portion of the tissues was fixed in 4% paraformaldehyde and subsequently embedded in paraffin.

### 2.2. Histomorphology and Immunohistochemical Analysis

Hematoxylin and eosin (H&E) and Picrosirius red staining were applied to 5-µm sections of paraffin-embedded liver tissue. All histological assessments were performed by a single investigator blinded to group allocation. The degree of steatosis and inflammatory activity was assessed semi-quantitatively using the non-alcoholic fatty liver disease activity score (NAS). NAS components (steatosis, 0–3; ballooning, 0–2; lobular inflammation, 0–3) were scored according to the NASH CRN system on H&E-stained sections from three randomly selected animals per group by an investigator blinded to group allocation. Hepatic fibrosis was assessed using histologic and molecular methods. Fibrosis extent was evaluated on Picrosirius red–stained liver sections by quantitative morphometric analysis of fibrotic area, as described above. In addition, hepatic expression of fibrosis-related genes (*Col1a1*, *Acta2*, and *Tgfb1*) was assessed by qRT-PCR. For Picrosirius red staining, sections were deparaffinized, rehydrated, and stained (Polysciences, Warrington, PA, USA) according to the manufacturer’s instructions to confirm collagen deposition.

Frozen liver sections were used for immunohistochemistry and Oil Red-O staining. Sections were washed for 10 min in 0.1 M PBS, and endogenous peroxidase activity was quenched with 3% hydrogen peroxide in PBS for 15 min. After rinsing with PBS, sections were incubated in a blocking solution containing 5% normal serum and 0.1% Triton X-100 for 30 min, then incubated overnight at 4 °C with a primary goat anti-CLEC4F/CLECSF13 polyclonal antibody (1:400; R&D Systems, Cat. No. AF2784, Minneapolis, MN, USA). A positive control (mouse liver tissue known to express CLEC4F in Kupffer cells) was included in each run to confirm antibody specificity. Sections were then incubated for 1 h with a biotinylated rabbit anti-goat IgG secondary antibody (1:200; Vector Laboratories, Newark, CA, USA; Cat. No. BA-5000, USA), followed by exposure to an avidin-biotin peroxidase complex (Vector Laboratories, Newark, CA, USA) for 1 h. Visualization was performed using a 0.05% DAB (3,3-diaminobenzidine tetrahydrochloride; Sigma, St. Louis, MO, USA) solution with hydrogen peroxide for 3–5 min. Primary antibody specificity was confirmed by omitting the primary antibody as a negative control. For Oil Red-O staining, frozen sections were fixed in 4% formaldehyde at room temperature for 10 min, washed with 60% isopropanol, and stained with 0.5% Oil Red O solution (Sigma) for 15 min. Nuclei were lightly counterstained with alum hematoxylin. Oil Red O-stained area was quantified using ImageJ software (Version 1.54g). For each animal, 3 random fields per 3 non-consecutive sections were captured at 100× magnification. The red-stained area was thresholded using the Color Deconvolution method with consistent threshold settings across all images. The percentage of Oil Red O-positive area relative to total tissue area was calculated for each field, and values were averaged per animal to yield a single data point per mouse.

### 2.3. Isolation of Peritoneal Macrophages

To obtain peritoneal macrophages, animals were injected intraperitoneally with 2 mL of 3% Brewer thioglycolate broth (Gibco, Carlsbad, CA, USA). After 72 h, animals were anesthetized with isoflurane and immediately sacrificed by cervical dislocation; isoflurane was used to minimize potential stress-induced macrophage activation prior to harvest, in accordance with institutional guidelines. An incision was made in the lower abdomen, and 8 mL of 2% inactivated fetal bovine serum (FBS) in PBS was injected into the abdominal cavity. The abdomen was massaged for 1 min, and lavage fluid was collected. Cells were washed by centrifugation at 300× *g* for 5 min (twice) and transferred to culture plates in RPMI-1640 medium containing 10% inactivated FBS.

### 2.4. RAW 264.7 Cell Culture

The murine macrophage cell line RAW 264.7 was obtained from the American Type Culture Collection (ATCC, Cat. No. TIB-71, Manassas, VA, USA) and maintained in RPMI-1640 medium supplemented with 10% FBS and 1% penicillin–streptomycin at 37 °C in a humidified 5% CO_2_ incubator. Cells were passaged at 70–80% confluence and used for experiments at low passage numbers.

### 2.5. Cell Viability Assay (WST-1)

Cell viability was assessed using the WST-1 assay (Roche, Basel, Switzerland) according to the manufacturer’s instructions. Cells (5.0 × 10^4^ cells/well) were seeded in 96-well plates (Falcon, Corning Inc., Durham, NC, USA). Cells were pretreated with simvastatin or ezetimibe for 1 h before stimulation with lipopolysaccharide (LPS; *Escherichia coli* O111:B4, Sigma-Aldrich, St. Louis, MO, USA) and cultured for 24 h. WST-1 reagent (10 µL) was added to each well and incubated at 37 °C for 2 h. For background measurement, 10 µL WST-1 was added to 100 µL serum-free RPMI-1640 medium in cell-free wells. Absorbance was measured at 450 nm using a microplate reader (Biotek, Winooski, VT, USA). Background values were subtracted, and experimental groups were normalized to untreated control. Simvastatin and ezetimibe concentrations of 40 µM each were selected as the maximum concentrations that did not significantly reduce cell viability (≥90% viability) based on dose–response bar plot. (Figure S3).

### 2.6. LX-2 Cell Culture and Treatment

The human hepatic stellate cell line LX-2 was purchased from MilliporeSigma (Cat. No. SCC064, Burlington, MA, USA) and maintained in DMEM/F12 medium supplemented with 5% FBS and 1% penicillin–streptomycin at 37 °C in a humidified 5% CO_2_ atmosphere; a low-serum condition was used to preserve the quiescent phenotype of stellate cells. For experiments, cells were seeded in 6-well plates (4 × 10^5^ cells/well), grown to 70–80% confluence, pre-treated with simvastatin (10 µM), ezetimibe (10 µM), or their combination for 1 h, and then stimulated with recombinant human TGF-β1 (2 ng/mL) for 24 h before harvesting for qRT-PCR analysis and phase-contrast imaging.

### 2.7. RNA Extraction and Quantitative Real-Time PCR

Total RNA was extracted from frozen liver specimens or cultured cells using TRIzol reagent (Invitrogen, Carlsbad, CA, USA) or the RNeasy Mini Kit (Qiagen, Valencia, CA, USA) according to the manufacturers’ protocols. cDNA was synthesized from 1 µg of total RNA using the RT Premix kit (iNTRON Biotechnology, Seongnam, Gyeonggi-do, Republic of Korea). Quantitative real-time PCR (qRT-PCR) was conducted on a QuantStudio 6 Flex system (Applied Biosystems, Foster City, CA, USA) using SYBR Green PCR Master Mix (Applied Biosystems) and gene-specific primers (Table 1). Cycle threshold (Ct) values were normalized against GAPDH as the endogenous reference, and relative mRNA expression levels were determined using the 2^−ΔΔCt^ comparative method.

### 2.8. Western Blotting

Protein lysates were prepared from cultured cells or mouse liver tissues using RIPA lysis buffer (Elpis-Biotech, Daejeon, Republic of Korea) supplemented with protease inhibitor cocktail and phosphatase inhibitor cocktail (Roche Diagnostics GmbH, Mannheim, Germany). Protein concentrations were quantified using a bicinchoninic acid (BCA) assay kit (Pierce, Appleton, WI, USA). Equal amounts of protein were resolved on 10–15% SDS-PAGE gels and electro-transferred onto PVDF membranes (Millipore, Carrigtwohill, Ireland). Membranes were blocked for 1 h at room temperature in TBST supplemented with 5% skim milk and subsequently incubated overnight at 4 °C with appropriate primary antibodies. Following three washes with TBST, membranes were incubated for 1 h at room temperature with HRP-conjugated secondary antibodies (Table S1). Protein bands were visualized using ECL detection reagent (GE Healthcare, Buckinghamshire, UK), and signals were captured with a Chemi-Doc imaging system (Bio-Rad, Hercules, CA, USA). Due to an accidental loss during the experiment, the complete membrane image for TNF-α could not be obtained. Consequently, quantitative interpretation of TNF-α protein data is limited, and conclusions regarding TNF-α are primarily supported by mRNA-level evidence, which is acknowledged as a methodological limitation.

### 2.9. Nuclear and Cytoplasmic Protein Extraction

Nuclear and cytoplasmic fractions were prepared using NE-PER Nuclear and Cytoplasmic Extraction Reagents (Thermo Fisher Scientific, Waltham, MA, USA) following the manufacturer’s instructions. For the cytoplasmic fraction, cells were pelleted at 500× *g* for 5 min, washed with ice-cold PBS, and centrifuged. CER I reagent (100 µL) was added, vortexed vigorously for 15 s, and incubated on ice for 10 min, followed by addition of CER II reagent (5.5 µL). After centrifugation at maximum speed (~16,000× *g*) for 5 min, the cytoplasmic supernatant was transferred to a pre-chilled tube. For the nuclear fraction, the remaining pellet was resuspended in ice-cold NER reagent (50 µL), vortexed for 15 s every 10 min over 40 min, and centrifuged at maximum speed for 10 min to collect the nuclear protein supernatant.

### 2.10. Flow Cytometry

RAW 264.7 cells (6 × 10^5^ cells/well) were seeded in 6-well plates. After 16 h, cells were pretreated with 40 µM simvastatin, ezetimibe, or both drugs for 1 h and then stimulated with LPS (1 µg/mL) for 24 h. Fc receptors were blocked using TruStain FcX PLUS antibody (BioLegend, Inc., San Diego, CA, USA) for 10 min. FITC-labeled CD206 and PE-labeled iNOS2 antibodies (0.125 µg each; BioLegend, Inc.) were added and incubated on ice for 20 min. For intracellular iNOS staining, after surface staining, cells were fixed with 4% paraformaldehyde for 15 min at room temperature, permeabilized with 0.1% Triton X-100 for 10 min, and then stained with PE-labeled iNOS2 antibody (0.125 µg; BioLegend, Inc.). After washing twice with PBS, macrophage polarity was measured by BD FACSAria III (BD Biosciences, San Jose, CA, USA), and data were analyzed using FlowJo software 10.5.3 (BD Biosciences). The same LPS stimulation and drug treatment conditions (concentration, timing, and duration) were applied to both RAW 264.7 cells and peritoneal macrophages to ensure comparability of results across the two cell types.

### 2.11. Immunofluorescence Assay

Peritoneal macrophages were seeded on 4-well chamber slides (Thermo Fisher Scientific, Inc.) and pre-treated with 40 µM simvastatin, ezetimibe, or their combination for 1 h. Cells were then stimulated with LPS (1 µg/mL) for 30 min, fixed with 4% paraformaldehyde for 15 min at room temperature, permeabilized with 0.1% Triton X-100 (Sigma-Aldrich, St. Louis, MO, USA), and blocked with 5% normal donkey serum for 1 h at room temperature. Cells were incubated with anti-NF-κB p65 antibody (Cell Signaling Technology, Inc., Danvers, MA, USA) overnight at 4 °C, followed by anti-rabbit IgG Alexa Fluor 488 (Jackson ImmunoResearch, West Grove, PA, USA) for 1 h at room temperature. Nuclei were stained with VECTASHIELD Anti-fade Mounting Medium with DAPI (Vector Laboratories, Burlingame, CA, USA). Image quantification was performed using ImageJ software (NIH, Bethesda, MD, USA) by an investigator blinded to group allocation. Nuclear and cytoplasmic regions of interest (ROIs) were defined for each cell based on DAPI-stained nuclear boundaries. The nuclear-to-cytoplasmic NF-κB p65 fluorescence intensity ratio was calculated after background subtraction using a cell-free region as a reference. A minimum of 30 cells per condition were quantified across at least six independent experiments.

### 2.12. Statistical Analysis

All statistical analyses were performed using data from at least three independent biological replicates per group. Here, “biological replicate” refers to independent animals (for in vivo experiments) or independently performed cell culture experiments using separately isolated cell preparations (for in vitro experiments); “technical replicate” refers to repeated measurements from the same biological sample. The experimental unit was the individual animal for in vivo experiments and the independent experiment for in vitro experiments; for microscopy-based readouts, multiple fields per animal were averaged to yield one value per animal. Given the small sample sizes (*n* = 3–6 per group) and the inability to assume a normal distribution, non-parametric tests were used throughout. All multi-group datasets were first analyzed with the Kruskal–Wallis H test; when significant (*p* < 0.05), prespecified pairwise comparisons—each treatment group vs. the HFD+TAA group, and HFD+TAA vs. normal control—were performed using two-sided exact Mann–Whitney U tests, with Holm adjustment for multiple comparisons.

For flow cytometric quantification of macrophage polarization, the percentage of iNOS^+^ cells in each treatment group was normalized to the LPS-stimulated group of the same experiment (set as 100%) to account for inter-experimental variability, and normalized values were compared with the hypothetical value of 100 using the one-sample Wilcoxon signed-rank test. Data are presented as individual values with the median, unless otherwise indicated. Statistical analyses were conducted using GraphPad Prism 10 (GraphPad Software 10.6.1, San Diego, CA, USA). Image quantification was performed using ImageJ software (NIH, Bethesda, MD, USA). Adjusted *p* < 0.05 was considered statistically significant.

## 3. Results

### 3.1. Statin and Ezetimibe Reduce Hepatic Steatosis in the HFD-TAA Model

**Figure 1:**
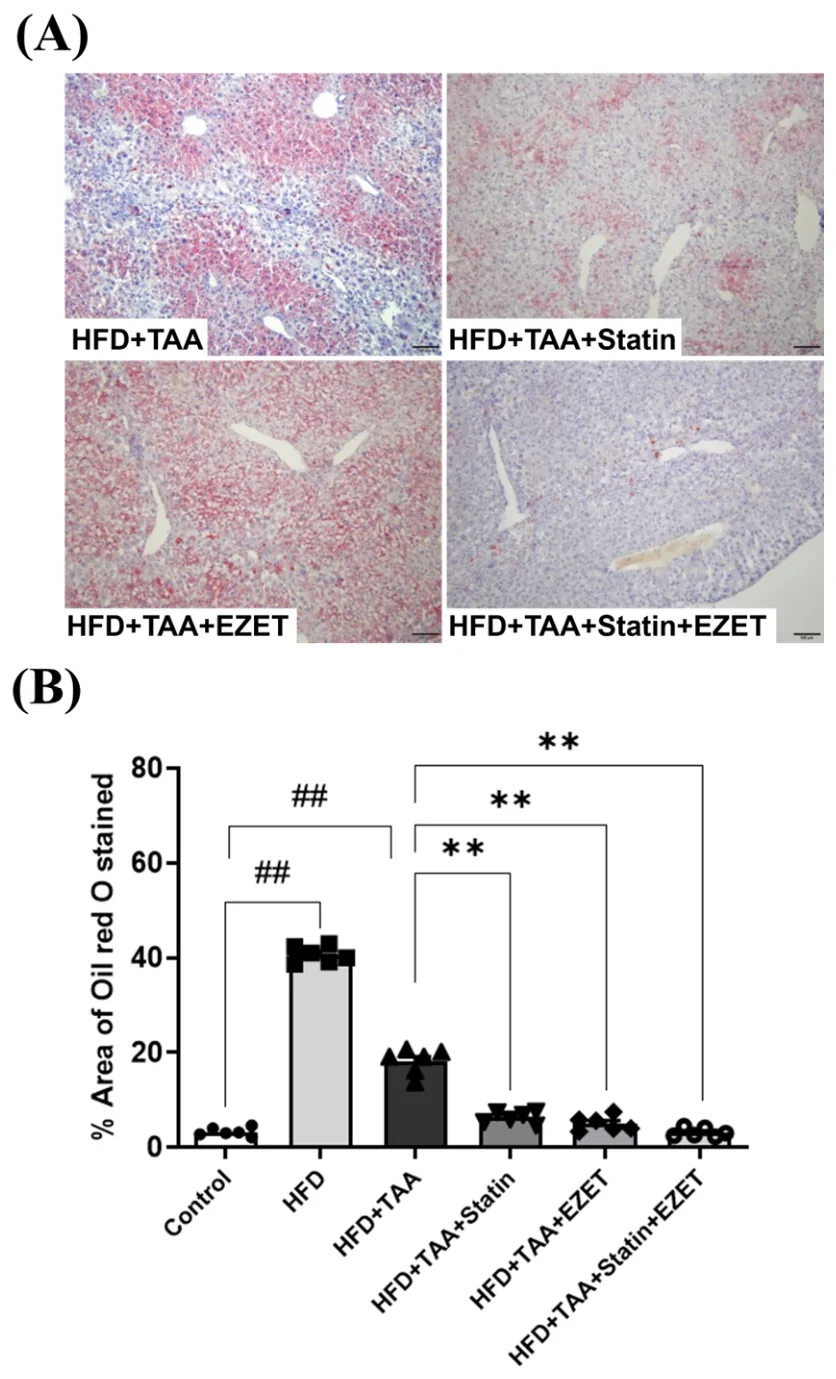
Statin and ezetimibe therapy reduce hepatic steatosis in the HFD-TAA murine model. (**A**) Representative Oil Red-O-stained liver sections (100×) from each experimental group. (**B**) Quantification of Oil Red O–positive area expressed as a percentage of total hepatic area. Each dot represents one animal (n = 6 per group); bars show mean ± SEM. Treatment effects were assessed by three prespecified comparisons versus HFD-TAA using exact Mann–Whitney U tests with Holm adjustment (adjusted *p* = 0.0066, 0.0044, and 0.0022 for +EZET, +Statin, and +Combination, respectively). Model-validation comparisons versus Control (exact Mann–Whitney U): HFD, *p* = 0.0022; HFD-TAA, *p* = 0.0022; +Statin, *p* = 0.0022; +EZET, *p* = 0.05; +Combination, *p* = 0.73. ** *p* < 0.01 vs. HFD-TAA; ## *p* < 0.01 vs. Control.

In both H&E and Oil Red-O staining, the HFD mouse group exhibited significantly greater steatosis (Figure S2). With additional TAA treatment, these groups showed histological MASH features. Increased hepatocyte volumes and greater accumulation of lipid droplets and inflammatory cells were observed in the HFD-TAA group; these effects were alleviated in the HFD-TAA-Statin, HFD-TAA-EZET, and HFD-TAA-Combination groups (Figure 1A).

Quantitative analysis with Oil Red-O staining showed that the Oil Red O-positive area was lower in the HFD-TAA group than in the HFD-alone group. This is an expected feature of TAA co-administration, which causes centrilobular hepatocellular injury and blunts hepatic lipid storage, mirroring the “burnt-out” phenotype of advancing human disease. Compared to the HFD-TAA control, a significantly lower percentage of stained area was further observed in the HFD-TAA-Statin, HFD-TAA-EZET, and HFD-TAA-Combination groups (Figure 1B). These findings indicated that statin and ezetimibe, as well as their combination, reduced hepatic steatosis. While combination therapy appeared to show a more favorable trend in improving steatosis, there was no statistically significant difference compared to either monotherapy group (HFD-TAA-EZET vs. HFD-TAA-Combination, *p* = 0.80; HFD-TAA-Statin vs. HFD-TAA-Combination, *p* = 0.79).

### 3.2. Statin and Ezetimibe Attenuate Hepatic Inflammation

**Figure 2:**
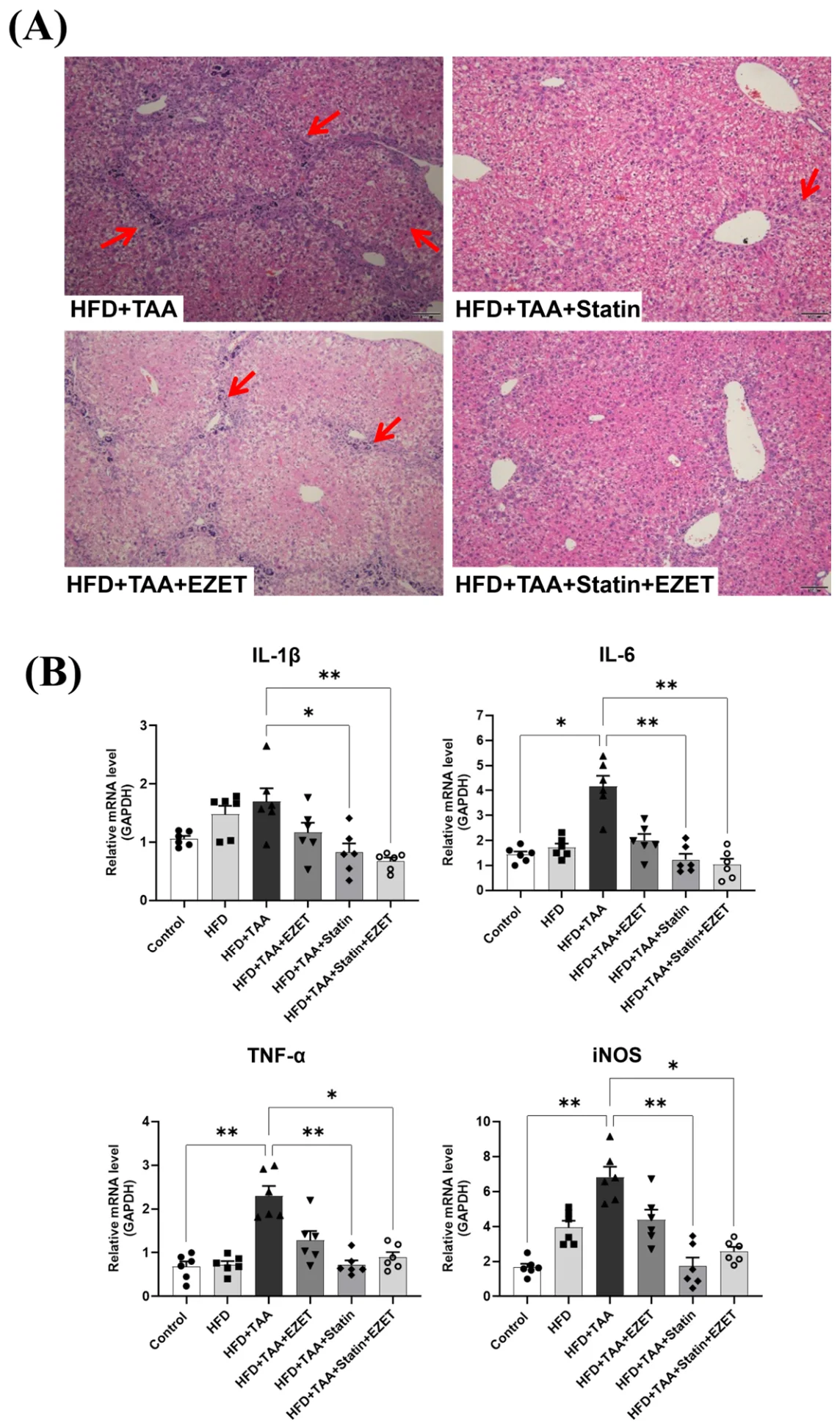
Statin and ezetimibe treatment attenuates hepatic inflammation in the HFD-TAA murine model. (**A**) Representative H&E-stained liver sections (100×). Red arrows indicate infiltration of polymorphonuclear and mononuclear inflammatory cells in portal and lobular areas. (**B**) Hepatic mRNA expression of pro-inflammatory cytokines (IL-1β, IL-6, TNF-α, and iNOS) assessed by qRT-PCR and normalized to GAPDH. Data are presented as mean ± SEM. Statistical comparisons were performed using the Kruskal–Wallis test followed by pairwise Mann–Whitney U tests. * *p* < 0.05, ** *p* < 0.01 versus the HFD-TAA group.

The HFD-TAA group exhibited pronounced inflammatory changes, including dense infiltration of polymorphonuclear and mononuclear inflammatory cells in the portal areas. These histological features were attenuated in mice treated with ezetimibe and further improved in the HFD-TAA-Statin, HFD-TAA-EZET, and HFD-TAA-Combination groups (Figure 2A). Histological inflammation scores based on the NASH CRN system are summarized in Figure S4; although a trend toward improvement was observed in treated groups, scores did not reach statistical significance, potentially reflecting the inherent sensitivity limitations of semi-quantitative histological scoring for subtle inflammatory changes.

Notably, molecular-level analysis provided complementary evidence of anti-inflammatory effects with greater sensitivity. Hepatic mRNA expression levels of pro-inflammatory markers, including IL-1β, IL-6, TNF-α, and iNOS, were quantified by qRT-PCR (Figure 2B). Compared to the HFD-TAA group, treatment with simvastatin or ezetimibe significantly reduced the expression of these inflammatory markers, with the HFD-TAA-Combination group showing the most prominent reduction in all four markers (*p* < 0.01 for each, compared with HFD-TAA); however, direct pairwise comparisons between the combination and either monotherapy did not reach statistical significance. The discrepancy between histological scores and molecular markers likely reflects the higher sensitivity of qRT-PCR in detecting early molecular changes that precede morphologically detectable tissue-level alterations.

### 3.3. Statin and Ezetimibe Reduce Hepatic Fibrosis

**Figure 3:**
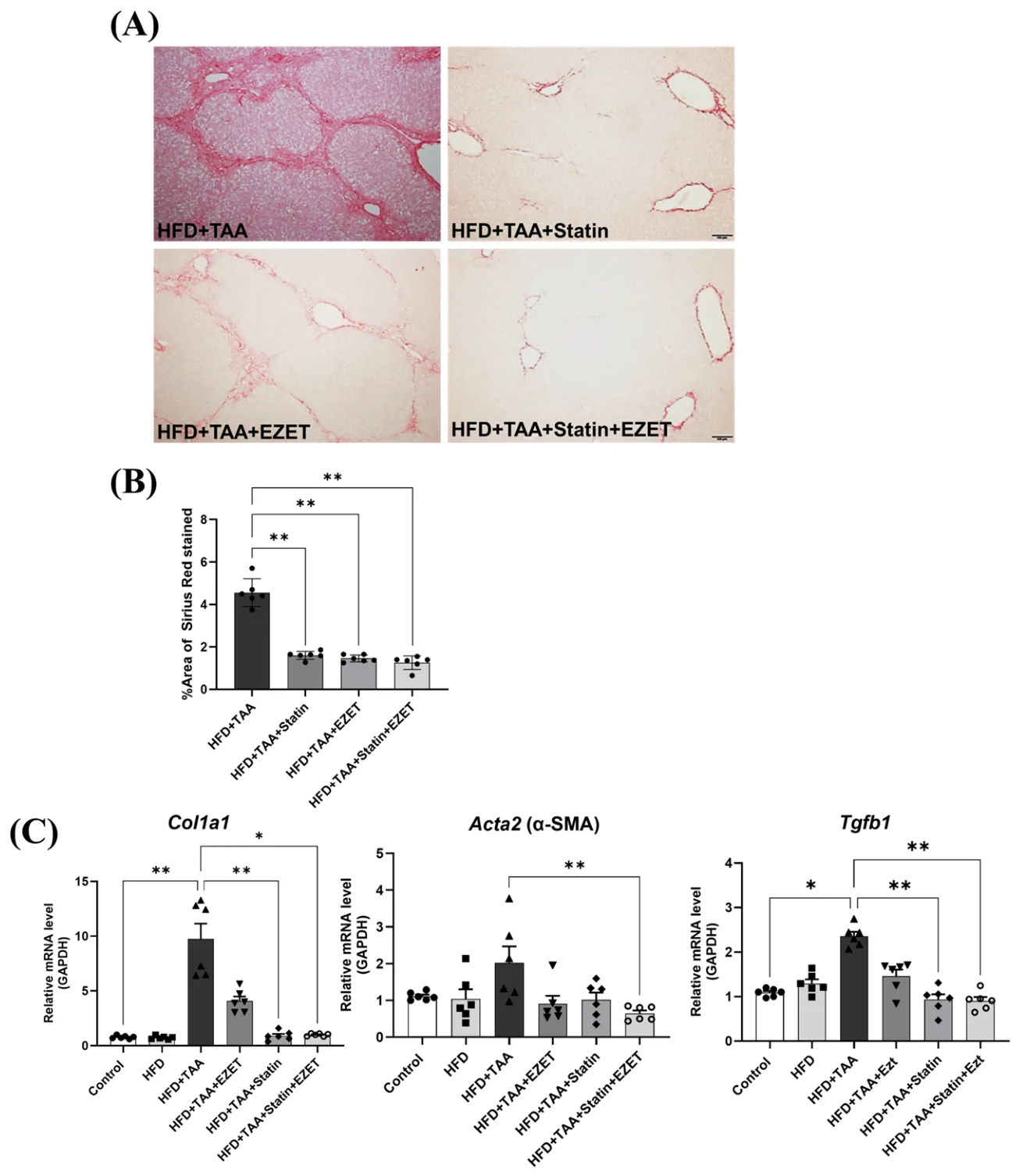
Combination therapy of statin and ezetimibe reduces hepatic fibrosis in the HFD-TAA murine model. (**A**) Representative Picrosirius Red-stained liver sections (100×) illustrating collagen deposition across experimental groups. (**B**) Quantification of Picrosirius red–positive area expressed as a percentage of total hepatic area. Each dot represents one animal (*n* = 6 per group); bars show mean ± SEM. Exact adjusted *p*-values: HFD-TAA vs. HFD-TAA+EZET, *p* = 0.0066; vs. HFD-TAA+Statin, *p* = 0.0044; vs. HFD-TAA+Statin+EZET, *p* = 0.0022. * *p* < 0.05, ** *p* < 0.01 versus the HFD-TAA group. (**C**) Hepatic mRNA expression of fibrogenic markers (*Col1a1*, *Acta2*/α-SMA, and *Tgfb1*/TGF-β1) assessed by qRT-PCR and normalized to GAPDH. Data are presented as mean ± SEM.

To examine whether statin and ezetimibe combination therapy influenced fibrosis, we conducted a quantitative assessment using Picrosirius red staining (Figure 3A). In the HFD-TAA group, prominent interlobular septation was observed, indicating advanced fibrosis. Treatment with ezetimibe (HFD-TAA-EZET group) resulted in a reduction in collagen deposition and partial improvement in fibrosis. The anti-fibrotic effect was more pronounced in both the HFD-TAA-Statin and HFD-TAA-Combination groups, with significantly decreased collagen fiber accumulation compared to the HFD-TAA group (*p* < 0.01 for each vs. HFD-TAA). Both groups exhibited notable amelioration of fibrosis, with the HFD-TAA-Combination group showing a numerically greater reduction in collagen deposition than the HFD-TAA-Statin group; however, this difference did not reach statistical significance in direct pairwise comparison and should be interpreted as a trend toward greater benefit rather than confirmed superiority. (Figure 3B).

In line with the histological findings, mRNA expression levels of fibrosis-associated markers, including collagen type I, α-smooth muscle actin (α-SMA), and transforming growth factor (TGF)-β1, were significantly elevated in the HFD-TAA group. Treatment with either simvastatin or ezetimibe suppressed these gene expressions, and the combination group exhibited the greatest numerical downregulation, although this was not statistically superior to monotherapy. Collagen type I and TGF-β1 were reduced in both the statin and combination groups, whereas α-SMA was significantly decreased only in the combination group; however, all treatment groups showed a trend toward lower expression compared with the HFD-TAA group. These findings suggest that both statin and ezetimibe inhibit liver fibrosis in this steatohepatitis model (Figure 3C).

### 3.4. Statin and Ezetimibe Suppress Hepatic Inflammatory Signaling

**Figure 4:**
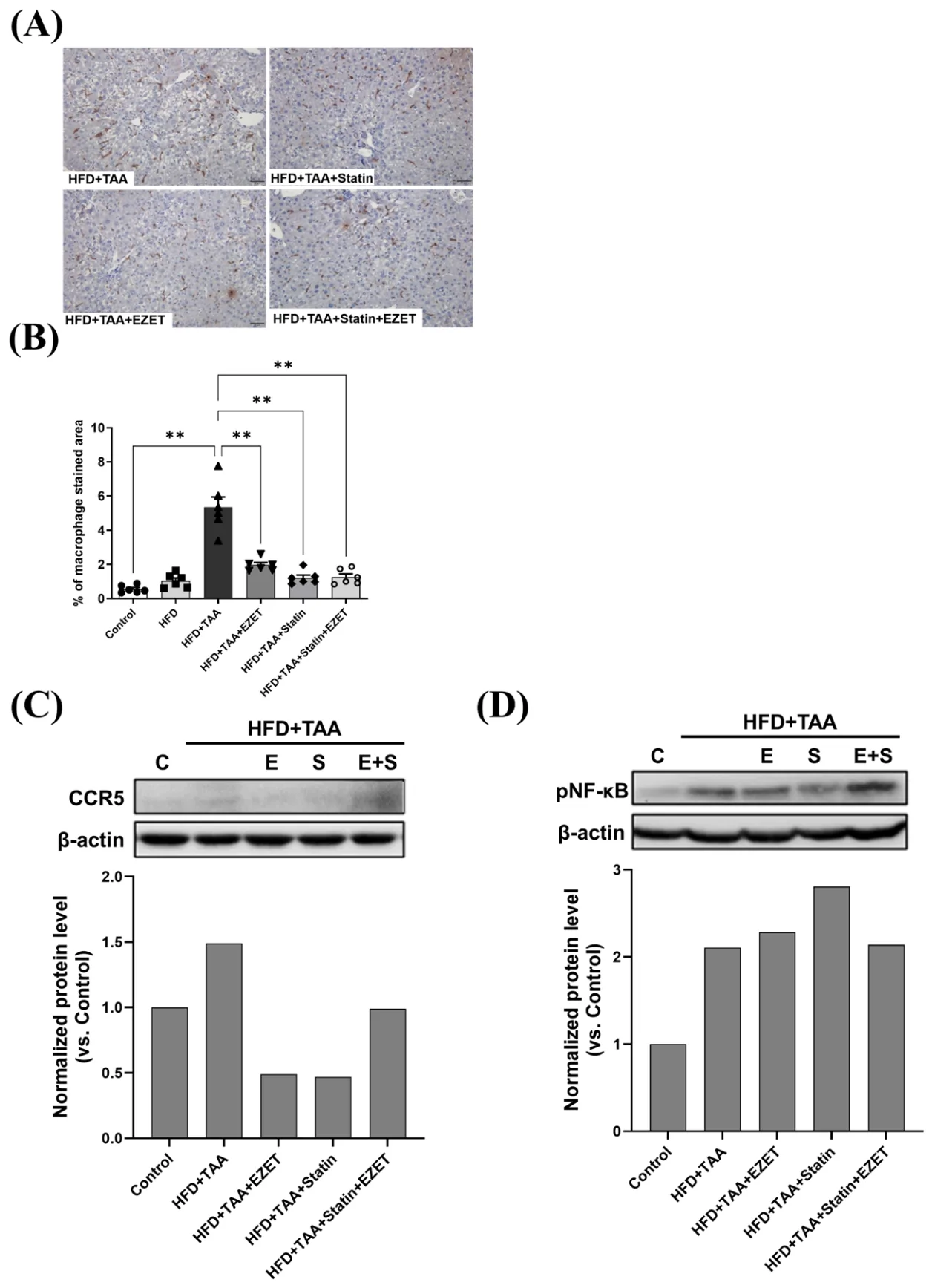
Statin and ezetimibe combination therapy reduces CLEC4F^+^ Kupffer cell abundance, with hepatic CCR5 and phospho-NF-κB p65 assessed descriptively. (**A**) Representative immunohistochemical images of liver sections stained for CLEC4F (hepatic resident macrophages; magnification 100×). (**B**) Quantification of the CLEC4F-positive area. (*n* = 6 animals per group; dots represent individual animals; midline, median). ** *p* < 0.01 vs. HFD+TAA (Kruskal–Wallis test followed by prespecified pairwise Mann–Whitney U tests with Holm adjustment (**C**) Representative Western blot of hepatic CCR5 with β-actin as the loading control (single experiment; densitometric values below are descriptive only, without statistical inference). (**D**) Representative Western blot of hepatic phospho-NF-κB p65 (Ser536) with β-actin (single experiment; densitometric values are descriptive only).

**Figure 5:**
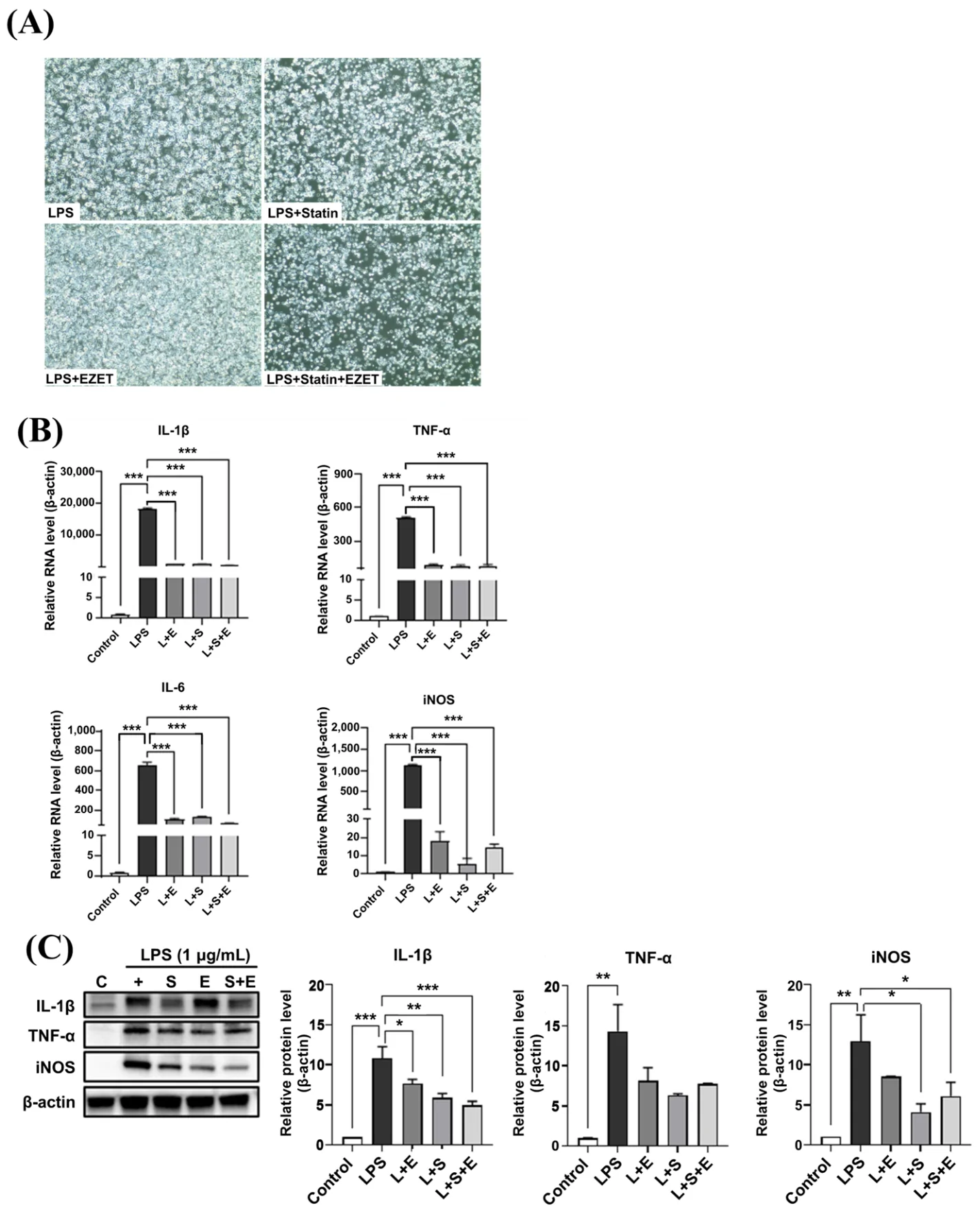
Statin and ezetimibe suppress LPS-induced macrophage activation and pro-inflammatory responses in vitro. (**A**) Representative phase-contrast images (200×) of RAW 264.7 macrophages stimulated with LPS (1 μg/mL) and treated with simvastatin, ezetimibe, or their combination. (**B**) mRNA expression of IL-1β, TNF-α, IL-6, and iNOS was assessed by qRT-PCR and normalized to GAPDH. (**C**) Western blot analysis of IL-1β, TNF-α, and iNOS protein levels with densitometric quantification normalized to β-actin. Data are presented as mean ± SEM. Cells were pretreated with simvastatin, ezetimibe, or both for 1 h prior to LPS stimulation. Statistical comparisons were performed using the Kruskal–Wallis test followed by pairwise Mann–Whitney U tests. * *p* < 0.05, ** *p* < 0.01, *** *p* < 0.001 versus indicated groups. ***Abbreviations***: *L*, *LPS*; *S*, *simvastatin*; *E*, *ezetimibe*.

In animal models, hepatic resident macrophages were identified by immunohistochemistry using a mouse CLEC4F/CLECSF13 antibody (Figure 4A). The number of Kupffer cells significantly increased in the HFD-TAA group. Immunohistochemical analysis showed a reduced percentage of CLEC4F-positive areas in the HFD-TAA-Statin, HFD-TAA-EZET, and HFD-TAA-Combination groups compared with HFD-TAA (Figure 4B), indicating a reduced abundance of CLEC4F^+^ resident Kupffer cells. Because CLEC4F identifies resident Kupffer cells without reporting their activation state or recruited populations, these findings were interpreted as correlative. C-C chemokine receptor 5 (CCR5) expression, quantified descriptively from a single membrane, was lower in the HFD-TAA-Statin, HFD-TAA-EZET, and HFD-TAA-Combination groups was also reduced in the HFD-TAA-Statin, HFD-TAA-EZET, and HFD-TAA-Combination groups (Figure 4C), indicating reduced hepatic inflammatory signaling. Phosphorylated NF-κB p65 (Ser536), assessed by Western blot in whole-liver lysates, was elevated in the HFD-TAA group; however, no consistent treatment-dependent change was discernible at the whole-tissue level (Figure 4D; single experiment, descriptive). Macrophage-specific NF-κB regulation may be diluted in whole-liver lysates, which consist predominantly of hepatocytes; macrophage-resolved analyses are presented in Figure 4 and Figure 5.

### 3.5. Statin and Ezetimibe Combination Inhibits LPS-Induced Macrophage Activation In Vitro

To investigate the anti-inflammatory mechanism of statin and ezetimibe in vitro, the murine macrophage cell line, RAW 264.7, was stimulated with LPS (1 µg/mL). Activated macrophages were treated with simvastatin (40 µM), ezetimibe (40 µM), or a combination of both. The mRNA levels of IL-1β, TNF-α, IL-6, and iNOS were significantly increased in LPS-treated cells compared to the control. Treatment with simvastatin, ezetimibe, or their combination markedly suppressed the LPS-induced upregulation of all four markers (Figure 5A,B). Western blot analysis confirmed a significant reduction in IL-1β and iNOS protein levels in the treated groups, especially in the combination group (Figure 5C). As noted in the Methods Section, complete immunoblot images for TNF-α could not be obtained due to an accidental loss during the experiment; consequently, conclusions regarding TNF-α are primarily supported by the robust mRNA-level evidence. These findings suggest that statin, ezetimibe, and combination therapy effectively inhibited RAW 264.7 macrophage activation and suppressed cytokine/inflammatory marker expression.

### 3.6. Flow Cytometric Analysis of Macrophage Polarization

**Figure 6:**
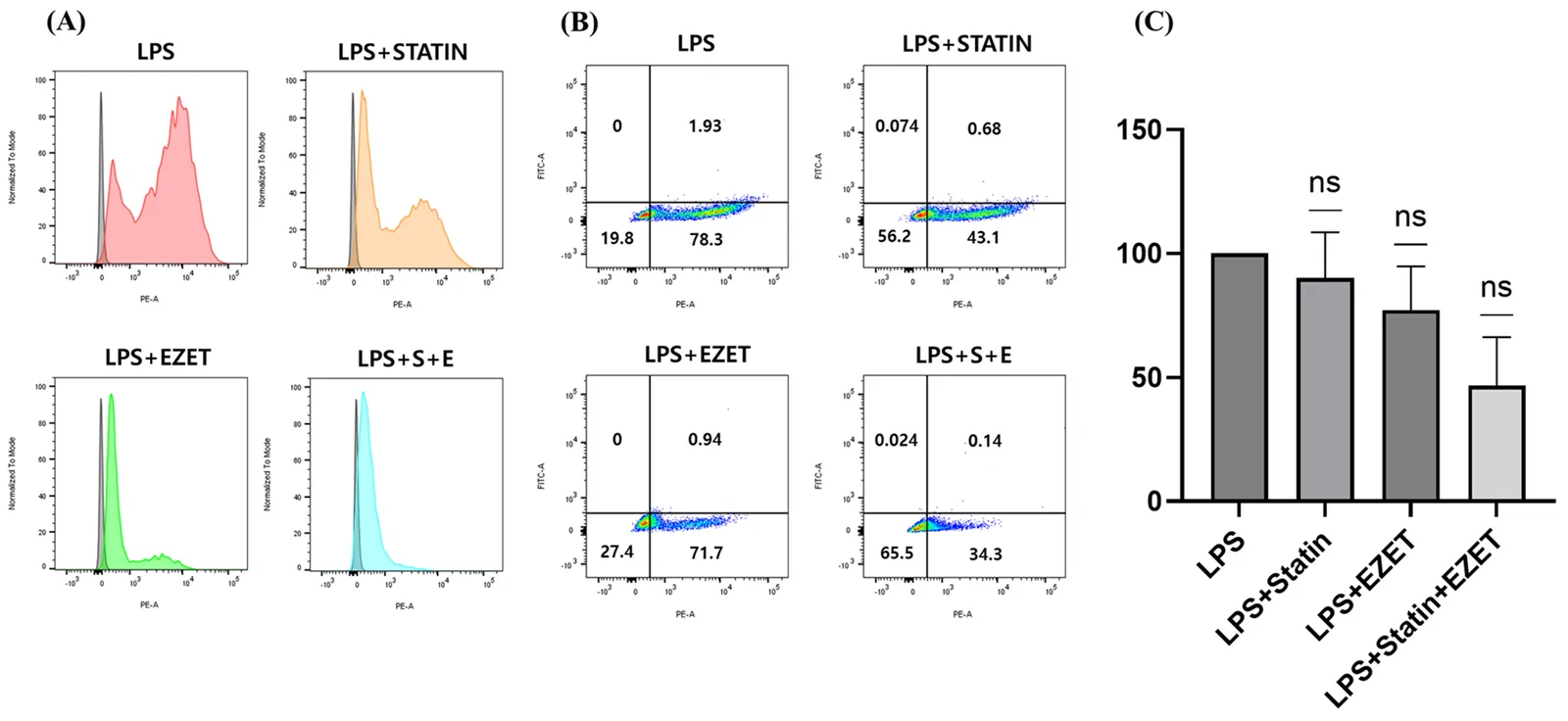
Statin and ezetimibe combination therapy suppresses LPS-induced M1 macrophage polarization in vitro. Flow cytometric analysis of macrophage polarization markers in RAW 264.7 macrophages stimulated with LPS (1 μg/mL) and treated with simvastatin, ezetimibe, or their combination. Cells were stained with PE-conjugated anti-iNOS (M1 marker) and FITC−conjugated anti-CD206 (M2 marker) antibodies. (**A**) Representative histograms of PE-iNOS fluorescence intensity. (**B**) Representative dot plots of FITC-CD206 versus PE-iNOS; numbers indicate the percentage of cells in each quadrant. The lower right quadrant (iNOS^+^/CD206^−^) represents the M1 macrophage population. (**C**) Quantification of iNOS^+^ (CD206^−^) macrophages from three independent experiments. Cells were pretreated with simvastatin, ezetimibe, or both for 1 h prior to LPS stimulation. Values were normalized to the LPS group of each experiment (100%). Data are presented as mean ± SEM. Statistical comparisons versus the hypothetical value of 100 (LPS) were performed using the one-sample Wilcoxon signed-rank test. ns, not significant. Data are representative of at least three independent experiments.

Flow cytometric analysis was performed using PE-conjugated anti-iNOS (M1 marker) and FITC-conjugated anti-CD206 (M2 marker) antibodies (Figure 6). In the LPS-only group, the proportion of iNOS-positive cells markedly increased to 78.3%, confirming effective M1 activation. Treatment with simvastatin or ezetimibe reduced the percentage of iNOS^+^ macrophages to 43.1% and 71.7%, respectively. The combination therapy group showed the most pronounced inhibition in the representative experiment, with only 34.3% of cells expressing iNOS. Notably, no detectable expression of the M2 marker CD206 was observed in any group, suggesting that the anti-inflammatory effects were attributable to the inhibition of M0-to-M1 activation rather than a phenotypic switch toward the M2 subtype. The absence of CD206 expression most likely reflects the enrichment of inflammatory monocyte-derived macrophages and/or relative depletion of resident CD206-expressing populations under strong LPS stimulation conditions; future studies should evaluate M2 marker expression at multiple time points and utilize additional M2 markers (e.g., Arg1, Fizz1) to better characterize polarization dynamics.

### 3.7. Statin and Ezetimibe Attenuate NF-κB Nuclear Translocation with Non-Significant MAPK Alterations

**Figure 7:**
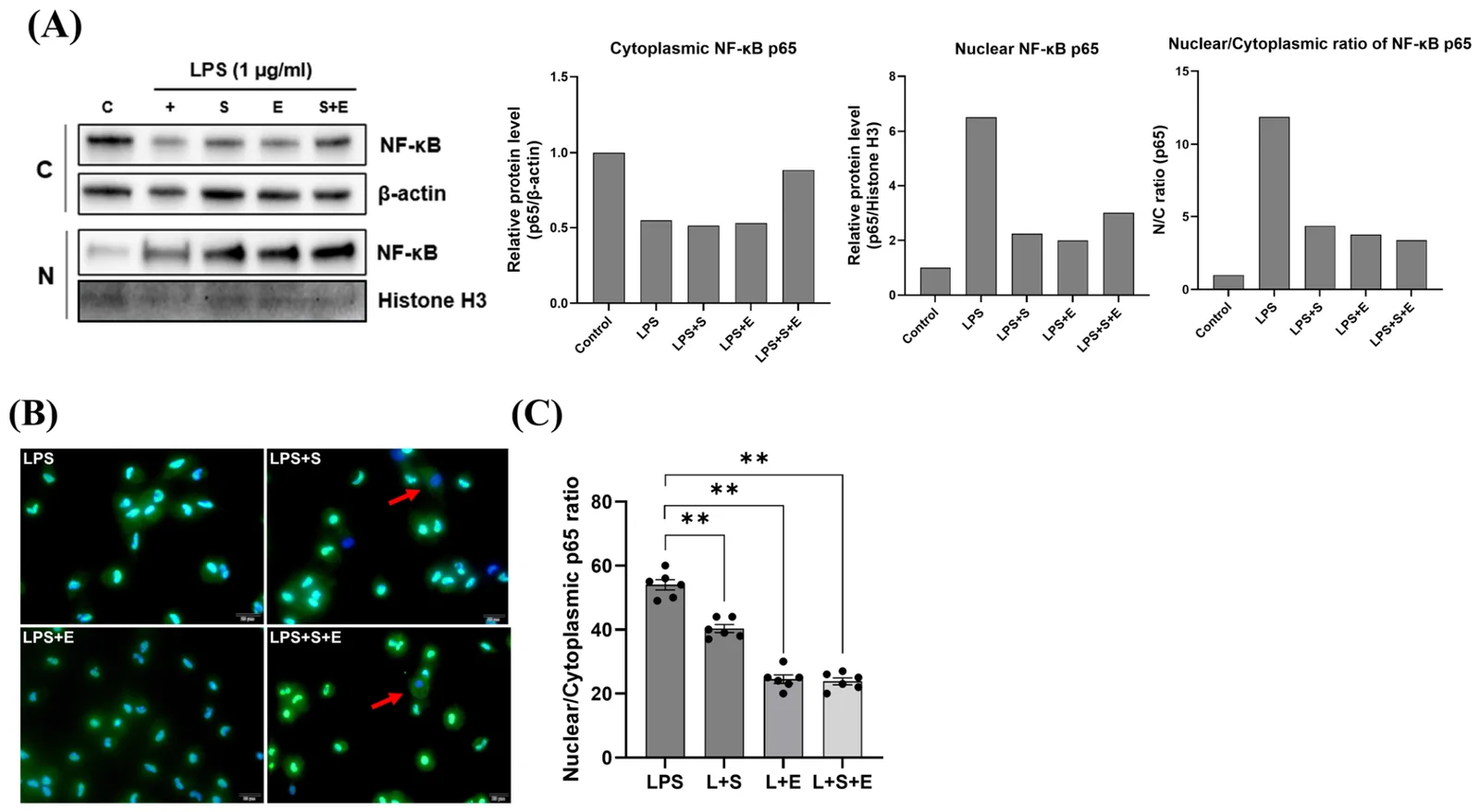
Statin and ezetimibe combination therapy inhibits the nuclear translocation of NF-κB in LPS-stimulated macrophages. (**A**) Western blot analysis of NF-κB in cytoplasmic (C) and nuclear (N) fractions of RAW 264.7 macrophages stimulated with LPS (1 μg/mL) and treated with simvastatin (S), ezetimibe (E), or their combination (S+E). β-actin and Histone H3 served as loading controls for the cytoplasmic and nuclear fractions, respectively. (**B**) Representative confocal immunofluorescence images of NF-κB p65 (green) N in thioglycolate-elicited peritoneal macrophages and DAPI-stained nuclei (blue) (100×). Cyan colocalization indicates nuclear translocation of p65; red arrows indicate cells with cytoplasmic NF-κB retention. (**C**) Nuclear-to-cytoplasmic ratio of NF-κB p65 fluorescence intensity in thioglycolate-elicited peritoneal macrophages. For each condition and experiment, ≥30 cells were quantified and averaged to yield a single value per experiment; each dot represents one independent experiment (*n* = 6). Bars show mean ± SEM. Prespecified comparisons versus LPS were performed using exact Mann–Whitney U tests with Holm adjustment (adjusted *p* = 0.0066, 0.0044, and 0.0022, respectively). ** *p* < 0.01 vs. LPS. Cells were pretreated with simvastatin, ezetimibe, or both for 1 h prior to LPS stimulation. ***Abbreviations***: *L*, *LPS*; *S*, *simvastatin*; *E*, *ezetimibe*.

Immunofluorescence staining of peritoneal macrophages demonstrated marked nuclear translocation of the NF-κB p65 subunit upon LPS stimulation (Figure 7B,C). Ezetimibe and combination therapy showed increased cytoplasmic localization of NF-κB, indicating inhibition of nuclear translocation. The nuclear/cytoplasmic fluorescence intensity ratio of p65 was significantly lower in the combination group compared to the LPS-only group (Figure 7C). Consistent with these findings, Western blot analysis of nuclear and cytoplasmic fractions in RAW 264.7 cells also demonstrated that the combination treatment exhibited the greatest numerical reduction in the nuclear-to-cytoplasmic ratio of NF-κB p65 (Figure 7A).

Analysis of downstream MAPK signaling (p38, JNK, ERK) showed non-significant trends toward reduction with ezetimibe and combination treatment (Figure S5), suggesting that under the present experimental conditions, the NF-κB pathway rather than MAPK signaling was the dominant mediator of the anti-inflammatory effects.

### 3.8. Statin and Ezetimibe Exert Limited Direct Effects on Hepatic Stellate Cells In Vitro

**Figure 8:**
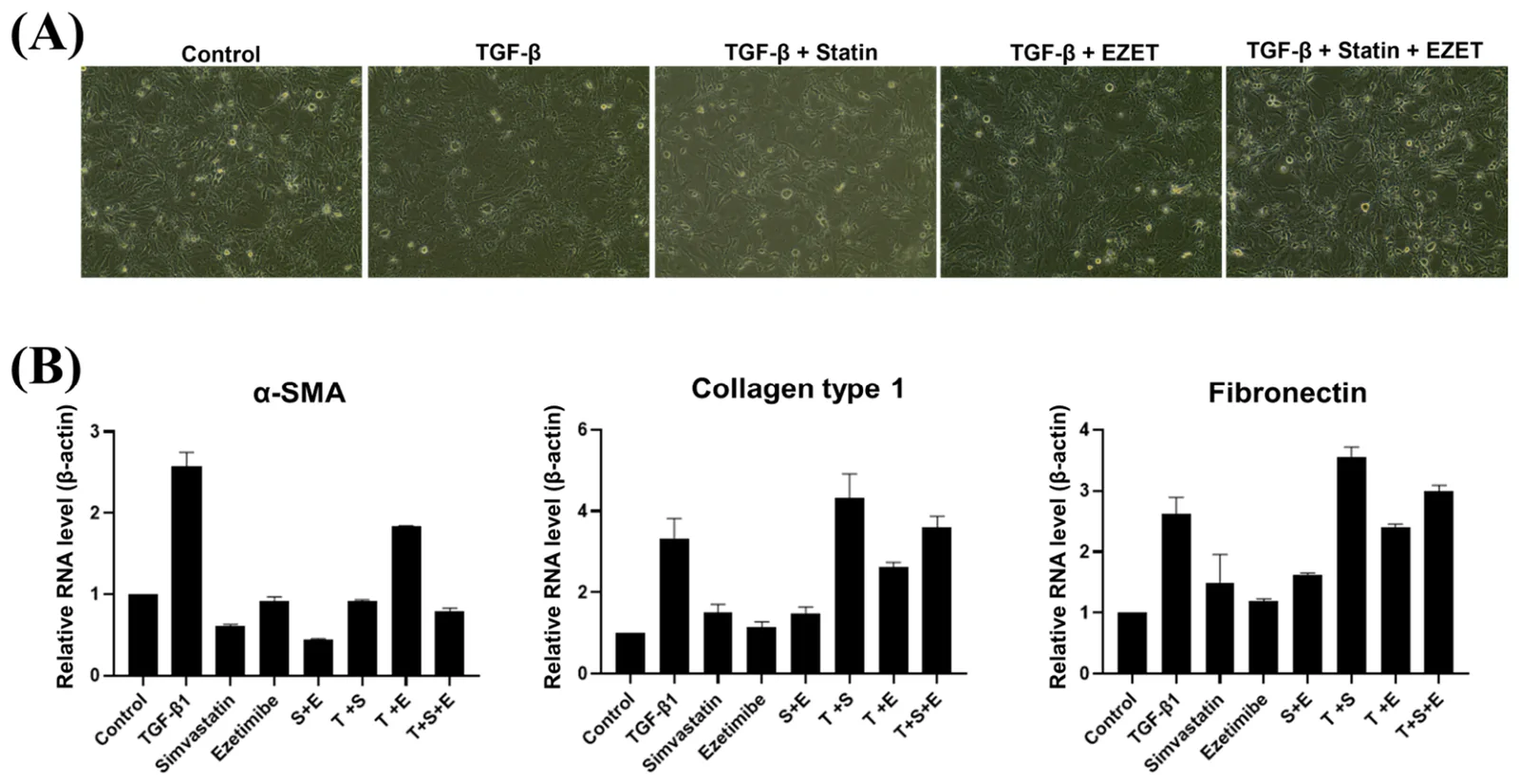
Statin and ezetimibe do not exert direct anti-fibrotic effects on TGF-β1-stimulated LX-2 human hepatic stellate cells. (**A**) Representative phase-contrast images of LX-2 cells under the indicated treatment conditions. Scale bar = 200 μm. (**B**) mRNA expression of fibrogenic markers (*ACTA2*/α-SMA, *COL1A1*/collagen type I, and *FN1*/fibronectin) assessed by qRT-PCR and normalized to β-actin. Cells were treated with TGF-β1 (2 ng/mL), simvastatin (10 μM), ezetimibe (10 μM), or their combinations as indicated. Data are presented as mean ± SEM of three independent experiments and statistical comparisons were performed using the Kruskal–Wallis test followed by pairwise Mann–Whitney U tests, consistent with the general statistical approach described in the Methods. ***Abbreviations***: *T*, *TGF-β1*; *S*, *simvastatin*; *E*, *ezetimibe*.

To evaluate whether statin and ezetimibe exert direct anti-fibrotic effects on hepatic stellate cells, the human hepatic stellate cell line, LX-2, was activated with TGF-β1 (2 ng/mL) in the presence or absence of the drugs. Morphologically, no notable differences were observed between the TGF-β1-stimulated groups and the treatment groups under phase-contrast microscopy. Quantitative analysis of fibrosis-associated markers, including *ACTA2* (α-SMA), *COL1A1* (collagen type I), and *FN1* (fibronectin) mRNA expression, showed no consistent or statistically significant inhibitory effects following treatment with simvastatin, ezetimibe, or their combination. These findings indicate that, under the present experimental conditions, statin and ezetimibe do not exert direct inhibitory effects on the TGF-β1-induced activation of LX-2 cells (Figure 8). This lack of direct efficacy on stellate cells further supports the notion that the in vivo anti-fibrotic effects of the combination therapy are likely contributed to indirectly through the modulation of macrophage activation.

## 4. Discussion

With the increasing global prevalence of MASLD, it is now estimated to affect approximately 32% of the adult population worldwide—pharmacological strategies that leverage existing, well-characterized agents are of particular translational interest. Following the 2024 FDA approval of resmetirom as the first MASH-specific agent, attention has turned to combination approaches and drug repurposing. While the anti-inflammatory and anti-fibrotic actions of statins are well documented, mechanistic data on the statin–ezetimibe combination in the specific context of MASH-associated hepatic inflammation and fibrosis remain limited.

Clinically, the ESSENTIAL randomized controlled trial demonstrated that ezetimibe (10 mg/day) combined with rosuvastatin (5 mg/day) significantly reduced hepatic fat by MRI-PDFF versus monotherapy (mean difference: −3.2%; *p* = 0.020), particularly in patients with insulin resistance and advanced fibrosis, suggesting a hepatoprotective benefit beyond lipid-lowering alone. However, that trial was not powered to evaluate changes in hepatic inflammation or fibrosis as primary endpoints, and the macrophage-level mechanism underlying the superior hepatic effect of the combination remained undefined. The present study directly addresses this mechanistic gap. Prior work by van Rooyen et al. employed a simple HFD-only model that generated minimal fibrosis, limiting anti-fibrotic signal detection; our HFD-TAA model overcomes this limitation by recapitulating both prominent steatosis and advanced fibrosis simultaneously, providing a multi-hit murine model of MASH with advanced fibrosis. Notably, while the HFD-TAA model exhibits lower absolute hepatic lipid storage (Oil Red O-positive area) compared to the HFD-alone model, this reflects the expected effects of TAA co-administration. TAA is bioactivated by CYP2E1, causing centrilobular hepatocellular injury that blunts weight gain and hepatic lipid storage [27,28,29]. Importantly, this reduction in steatosis alongside advancing fibrosis closely mirrors the “burnt-out” phenotype characteristic of progressing human MASH. Furthermore, while ezetimibe’s canonical mechanism involves NPC1L1 inhibition, NPC1L1-independent anti-inflammatory effects have been demonstrated in vitro and in atherosclerosis models, providing a mechanistic rationale for its inclusion despite species differences in hepatic NPC1L1 expression.

Macrophages play a pivotal role in the progression of hepatic fibrosis through multiple signaling pathways, including cytokine production and chemokine receptor activation. In particular, polarization toward the pro-inflammatory M1 phenotype drives fibrogenic signaling in the liver. In our study, statin–ezetimibe combination therapy reduced hepatic expression of CCR5 and iNOS, as evidenced by immunohistochemistry and flow cytometry. These findings suggest that the anti-inflammatory effects of the therapy are consistent with suppression of M1-like activation in cultured macrophages. The reduction in CCR5 expression in rodent hepatic tissue is consistent with decreased inflammatory signaling, which is known to adopt an M1-like phenotype under inflammatory conditions. Statins have been shown to downregulate chemokine receptors, including CCR2 and CCR5, in other disease models; our data extend these observations to the context of steatohepatitis.

While the statin–ezetimibe combination significantly reduced the proportion of iNOS-positive macrophages, indicative of suppressed M1 polarization, no detectable expression of the M2 marker CD206 was observed in any group (Figure 6). This finding suggests that the observed anti-inflammatory effects were not attributable to a phenotypic switch toward the M2 subtype, but rather to the inhibition of M0-to-M1 activation. The absence of CD206 expression most likely reflects an enrichment of inflammatory monocyte-derived macrophages and/or a relative depletion of resident CD206-expressing populations under the strong inflammatory stimulus employed (LPS, 1 µg/mL for 24 h). Alternatively, M2 polarization may require longer stimulation or distinct cytokine environments not fully recapitulated by LPS alone. Future studies should assess M2 markers at multiple time points and include additional markers—such as Arg1 and Fizz1—to more comprehensively characterize macrophage polarization dynamics. The reduction in iNOS expression with combination therapy is consistent with suppression of classical macrophage activation; whether the two agents interact additively or synergistically could not be determined in the present study.

Our findings also provide mechanistic evidence that the statin–ezetimibe combination inhibits macrophage activation through suppression of the NF-κB signaling cascade. We observed reduced nuclear translocation of the p65 subunit in two independent cultured macrophage models, providing subcellular-level evidence consistent with NF-κB pathway inhibition, thereby extending previous studies that inferred NF-κB suppression indirectly through downstream cytokine levels by providing direct subcellular localization evidence. Although ezetimibe exerts its canonical effects by inhibiting the NPC1L1 receptor, its precise role in liver fibrosis remains unclear given species differences in hepatic NPC1L1 expression. Using the HFD-TAA model, we demonstrate concurrent attenuation of inflammation and fibrosis and provide mechanism-level evidence that the therapeutic benefit is consistent with macrophage-associated mechanisms—characterized by inhibition of NF-κB nuclear translocation and suppression of M1 polarization—rather than a direct effect on stellate cell activation.

Despite these promising findings, several limitations must be acknowledged. First, while our high-fat diet and thioacetamide (HFD-TAA) murine model effectively recapitulates the histological, inflammatory, and fibrotic features of human MASH, several inherent model and experimental limitations must be noted. Systemic metabolic parameters—including body weight trajectories, glucose homeostasis, and serum lipid profiles—were not assessed. Consequently, the HFD-TAA model reflects a diet- and toxin-induced steatohepatitis rather than a purely metabolically driven MASH, necessitating caution when extrapolating these anti-inflammatory and antifibrotic findings to clinical settings. Additionally, our study lacked a separate baseline time point to histologically confirm fibrosis establishment prior to treatment initiation; although TAA-induced fibrosis at the utilized dose and duration is well-documented, direct baseline verification would have further strengthened the experimental design. Second, regarding the combination effect, while all three treatment groups showed improvement, the numerical superiority of combination therapy over monotherapy did not reach statistical significance in direct pairwise comparisons.

Accordingly, the combination benefit should be interpreted as directionally consistent and greater rather than definitively synergistic. Relatedly, histological inflammation scores did not reach statistical significance despite significant reductions in molecular inflammatory markers (IL-1β, IL-6, TNF-α, iNOS), likely reflecting the limited sensitivity of semi-quantitative histological scoring compared to qRT-PCR. Our conclusions regarding anti-inflammatory effects therefore rely primarily on molecular rather than histological evidence. Similarly, the non-significant trends in MAPK signaling (p38, JNK, ERK) should not be interpreted as definitive exclusion of MAPK pathway involvement; rather, our data indicate that the NF-κB pathway was the dominant signaling axis under the present experimental conditions. Furthermore, the sample sizes used in some experiments were relatively small (*n* = 3–6 per group), which may limit statistical power and the robustness of certain comparisons. Mice were group-housed and cage identifiers were not retained; therefore, cage-level effects could not be modeled. Although non-parametric statistical tests were applied to account for the small sample sizes and non-normal distributions, these findings should be confirmed in larger cohorts with a priori power analysis. Third, our in vivo readouts are not macrophage-specific: CLEC4F reports resident Kupffer cell abundance rather than activation and does not distinguish resident from recruited populations, and whole-liver CCR5 and p-NF-κB reflect all hepatic cell types. Conversely, RAW 264.7 cells and thioglycolate-elicited peritoneal macrophages are cultured macrophage models and not hepatic Kupffer cells. The macrophage-related mechanism proposed here therefore represents an association rather than a demonstrated macrophage-specific mechanism. Relatedly, while our data are consistent with a macrophage-mediated anti-fibrotic mechanism, direct evidence linking macrophage inhibition to stellate cell quiescence was not obtained; definitive testing will require analyses of isolated hepatic macrophages, macrophage–stellate cell co-culture systems, or in vivo macrophage depletion studies. Additionally, the M1/M2 characterization was limited to iNOS and CD206 only, and no M2-positive control was included; future studies should employ additional polarization markers (e.g., Arg1, Fizz1) and include appropriate controls. The TNF-α Western blot data should be interpreted cautiously, as the complete membrane image could not be obtained due to an accidental loss during the experiment; TNF-α conclusions are therefore primarily supported by the robust mRNA-level evidence. Additionally, cell viability in LX-2 cells was not formally assessed by a quantitative assay; the 10 µM concentration was selected based on prior reports of preserved viability at this dose, and no morphological evidence of cytotoxicity was observed by phase-contrast microscopy. Cell viability at the combined concentration (40 + 40 µM) in RAW 264.7 cells was not directly assessed; therefore, no viability claim is made for the combination condition. Fourth, a limitation of the in vitro experiments is that drugs were administered prior to LPS stimulation, modeling prevention of macrophage activation rather than treatment of already-present Kupffer cells. While this pre-treatment paradigm is mechanistically appropriate for investigating mevalonate pathway-dependent NF-κB inhibition, as statins exert their anti-inflammatory effects primarily through upstream blockade of the mevalonate pathway, the therapeutic implications for treating established macrophage activation require further investigation. Lastly, this study focused primarily on the NF-κB pathway; MASH pathogenesis is multifactorial, and further investigation into how this drug combination modulates other critical pathways—such as endoplasmic reticulum stress, mitochondrial dysfunction, and oxidative stress—will provide a more comprehensive understanding of its therapeutic breadth. Fifth, pharmacokinetic and pharmacodynamic assessments were not performed. Plasma and hepatic drug exposure were not measured, and target engagement (e.g., HMG-CoA reductase inhibition, NPC1L1 occupancy) was not verified; dose selection was therefore based on published rodent studies rather than exposure-matched translation of clinical doses. Pharmacokinetic and pharmacodynamic analyses should be incorporated in future studies to optimize dosing regimens for human translation.

Taken together, the mechanistic data presented here carry direct translational significance. The statin–ezetimibe combination suppresses macrophage NF-κB activation—a pathway positioned upstream of the pro-inflammatory cytokine cascade (IL-1β, IL-6, TNF-α) that drives hepatocyte injury, Kupffer cell recruitment, and stellate cell fibrogenic activation. Whether the observed anti-inflammatory and antifibrotic effects are independent of lipid lowering could not be determined in the present study. This mechanistic hierarchy provides a plausible biological explanation for the superior hepatic fat reduction observed in the ESSENTIAL trial and suggests that the combination’s hepatic benefits may extend to inflammatory and fibrotic endpoints that short-duration clinical trials are underpowered to detect. The absence of direct stellate cell suppression in our LX-2 model is consistent with the anti-fibrotic benefit being largely downstream of macrophage suppression. Prospective clinical trials incorporating liver biopsy endpoints or validated non-invasive markers of hepatic inflammation—such as circulating IL-1β, IL-6, or NF-κB pathway-associated biomarkers assessed alongside MRI-PDFF and magnetic resonance elastography—are needed to validate these observations in patients with MASH and concurrent dyslipidemia. Given its well-established safety profile in large cardiovascular trials and proven cost-effectiveness, the statin–ezetimibe combination represents a highly viable candidate for drug repurposing in MASH management. This study demonstrates that statin–ezetimibe combination therapy attenuates concurrent hepatic inflammation and fibrosis in a multi-hit MASH mouse model, at least in part through macrophage-associated NF-κB inhibition, with anti-fibrotic effects consistent with an indirect mechanism—mediated through macrophage suppression rather than direct hepatic stellate cell inhibition. These mechanistic findings are consistent with the clinical hepatic fat reduction observed in the ESSENTIAL randomized trial and provide a plausible mechanistic framework linking this regimen to hepatic inflammatory and fibrotic endpoints. Macrophage NF-κB signaling is identified as a pharmacologically tractable target within the statin–ezetimibe combination framework, positioning this widely used, clinically established regimen as a compelling drug-repurposing candidate in the emerging era of MASH pharmacotherapy. Future validation studies should incorporate pharmacokinetic confirmation of drug exposure, larger cohort sizes with a priori power analysis, and human-relevant models, such as hepatic organoids or ex vivo liver tissue, to bridge the translational gap between these preclinical findings and clinical application in MASH patients with concurrent dyslipidemia.

## Figures and Tables

**Table 1:** List of real-time qPCR primer sequences.

Gene	Direction	Sequence (5′→3′)	Product Length (bp)
**α-SMA (*Acta2*)**	F	5′-CCTTCGTGACTACTGCCGAG-3′	235
	R	5′-GTTTCGTGGATGCCCGCTG-3′	
***Tgfb1***	F	5′-ATTCCTGGCGTTACCTTGG-3′	120
	R	5′-AGCCCTGTATTCCGTCTCCT-3′	
***Cola1***	F	5′-TGACTGGAAGAGCGGAGAGT-3′	117
	R	5′-GACGGCTGAGTAGGGAACAC-3′	
***Pdgfd***	F	5′-CAATTCGGACTAGAGGAAGCAG-3′	238
	R	5′-CTTCCGGTTGGAAATCTTCCAC-3′	
***Pparg***	F	5′-CACAATGCCATCAGGTTTGG-3′	82
	R	5′-GCTGGTCGATATCACTGGAGATC-3′	
***Tnf***	F	5′-GGCATGGATCTCAAAGACAACC-3′	240
	R	5′-CAGGTATATGGGCTCATACCAG-3′	
***Il1b***	F	5′-CATCCAGCTTCAAATCTCGCAG-3′	212
	R	5′-CACACACCAGCAGGTTATCATC-3′	
***Il6***	F	5′-CATGTTCTCTGGGAAATCGTGG-3′	210
	R	5′-GTACTCCAGGTAGCTATGGTAC-3′	
**β-actin (*Actb*)**	F	5′-TTGCTGACAGGATGCAGAAG-3′	141
	R	5′-ACATCTGCTGGAAGGTGGAC-3′	

## Data Availability

All data generated or analyzed during this study are included in this published article and Supplementary data. The underlying raw data are available from the corresponding author upon reasonable request.

## Supplementary Materials

The following supporting information can be downloaded at: https://www.mdpi.com/article/10.3390/cells15181700/s1, Figure S1. Experimental design and treatment timeline for the HFD-TAA MASH mouse model; Figure S2. Histological confirmation of established MASH prior to treatment initiation at week 6; Figure S3. Dose-response effects of simvastatin and ezetimibe on macrophage cell viability; Figure S4. Semi-quantitative histological scoring of hepatic activity using the NASH CRN system; Figure S5. Statin and ezetimibe combination therapy does not significantly attenuate the MAPK signaling pathway in LPS-stimulated RAW 264.7 macrophages; Table S1. Specifications of primary and secondary antibodies used in Histomorphology and Immunohistochemical Analysis.

## Abbreviations

CCR5	C-C chemokine receptor 5
COL1A1	collagen type I
CER	Cytoplasmic Extraction reagents
H&E	Hematoxylin and eosin
HFD	High-fat diet
iNOS	inducible nitric oxide synthase
IL	Interleukin
LDL	low-density lipoprotein
LPS	lipopolysaccharide
MASH	Metabolic-dysfunction associated steatohepatitis
MASLD	Metabolic-dysfunction associated steatotic liver disease
MAPK	Mitogen-activated protein kinase
NPC1L1	Niemann-Pick C1-Like1
NOS	Nitric oxide synthase
NASH	Nonalcoholic steatohepatitis
NASH CRN	Non-alcoholic steatohepatitis clinical Research Network
NF-κB	Nuclear factor kappa-light-chain-enhancer of activated B cells
PBS	Phosphate-buffered saline
PE	Phycoerythrin
PCR	polymerase chain reaction
PI3K	Phosphoinositide 3-kinase
ROS	reactive oxygen species
RNA	Ribonucleic acid
SMA	Smooth muscle actin
SLD	Steatotic liver disease
SDS-PAGE	sulfate-polyacrylamide gel electrophoresis
TAA	Thioacetamide
TGF	Transforming growth factor
TNF	Tumor necrosis factor
T2DM	type 2 diabetes mellitus
WST-1	Water-Soluble Tetrazolium salt-1
