# Supplementary material for: Statin–Ezetimibe Attenuates Hepatic Inflammation and Fibrosis in a Diet- and Toxin-Induced Steatohepatitis Model, Associated with Macrophage NF-κB Inhibition
Source: Cells. 2026 Sep 19;15(18):1700. doi: 10.3390/cells15181700 (PMC13605004; doi:10.3390/cells15181700)
Supplement: Supplementary file 1 [file cells-15-01700-s001.zip › cells-4546690-supplementary.pdf]

# List of Supplementary Data

Figure S1. Experimental design and treatment timeline for the HFD-TAA MASH mouse model.

Figure S2. Histological confirmation of established MASH prior to treatment initiation at week 6.

Figure S3. Dose-response effects of simvastatin and ezetimibe on macrophage cell viability.

Figure S4. Histological evaluation of hepatic inflammation and steatosis using the NASH CRN scoring system.

Figure S5. Statin and ezetimibe combination therapy does not significantly attenuate the MAPK signaling pathway in LPS-stimulated RAW 264.7 macrophages.

Table S1. Specifications of primary and secondary antibodies used in Histomorphology and Immunohistochemical Analysis

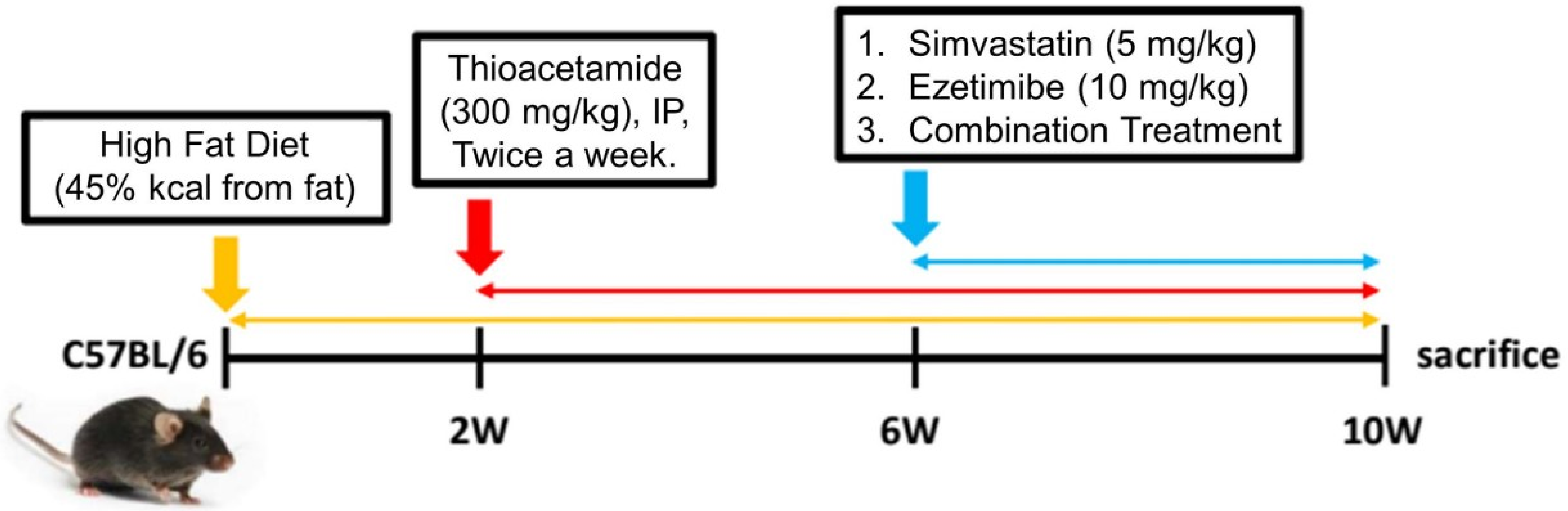


**Figure S1. Experimental design and treatment timeline for the HFD-TAA MASH mouse model.** Male C57BL/6 mice (7 weeks old) were maintained on a high-fat diet (HFD; 45% kcal from fat) from week 0 to week 10. Thioacetamide (TAA; 300 mg/kg) was administered via intraperitoneal injection twice weekly from week 2 to week 10 to induce hepatic inflammation and fibrosis. Pharmacologic treatment with simvastatin (5 mg/kg), ezetimibe (10 mg/kg), or their combination was initiated at week 6 and continued for 4 weeks until sacrifice at week 10.

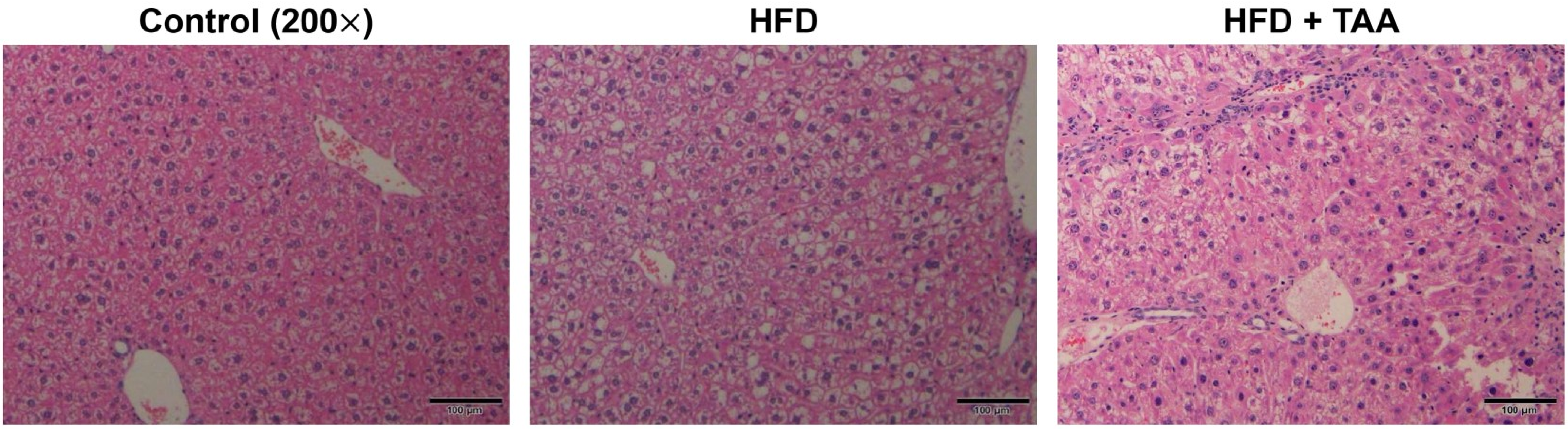


**Figure S2. Histological confirmation of established MASH prior to treatment initiation at week 6.** Representative H&E-stained liver sections from a separate pilot cohort (n = 3 per group) evaluating normal diet control (Control), HFD alone, and HFD with TAA (HFD-TAA) groups at the 6-week time point (4 weeks after TAA initiation). The HFD-TAA group demonstrates characteristic MASH features, including pronounced macrovesicular steatosis, hepatocellular ballooning, and lobular inflammatory cell infiltration, confirming that established steatohepatitis was present at the time pharmacologic therapy was initiated. These qualitative observations were used to verify disease establishment prior to the main experiment and were not quantified. Magnification: 200×. Scale bar: 100 μm.

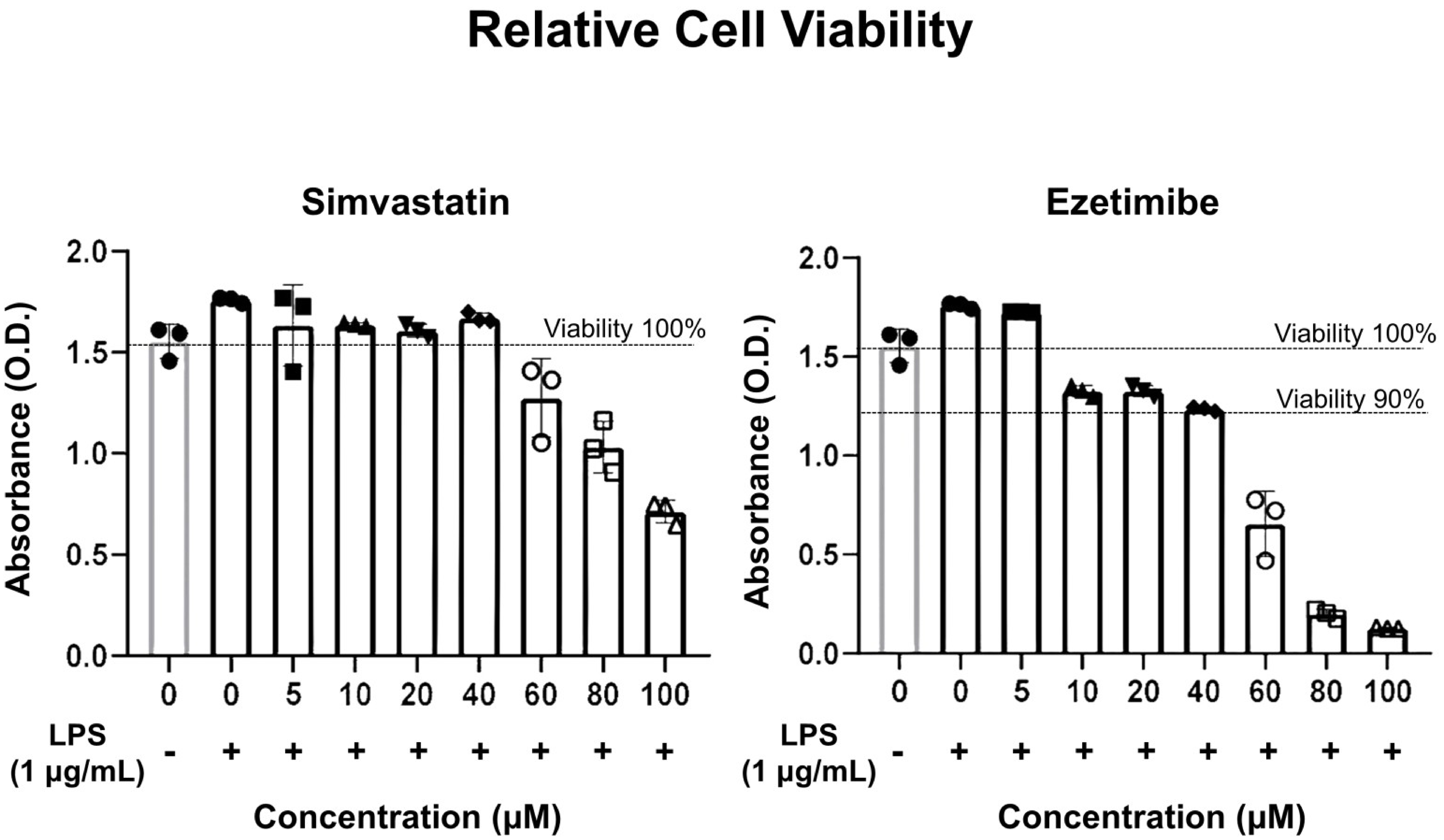


**Figure S3. Dose-response effects of simvastatin and ezetimibe on macrophage cell viability.** Cell viability was evaluated using the WST-1 assay. Macrophages were pretreated with various concentrations (0 to 100 μM) of either simvastatin (left panel) or ezetimibe (right panel) for 1 hour, followed by stimulation with lipopolysaccharide (LPS, 1 μg/mL) for 24 hours. Relative cell viability is presented as optical density (O.D.) measured at 450 nm. The upper and lower dashed lines indicate the 100% and 90% viability thresholds, respectively, relative to the untreated control. Based on these dose-response graphs, a concentration of 40 μM for both simvastatin and ezetimibe was selected for subsequent *in vitro* experiments, as it represents the maximum dose that effectively maintains cell viability at or above 90% without inducing significant cytotoxicity.

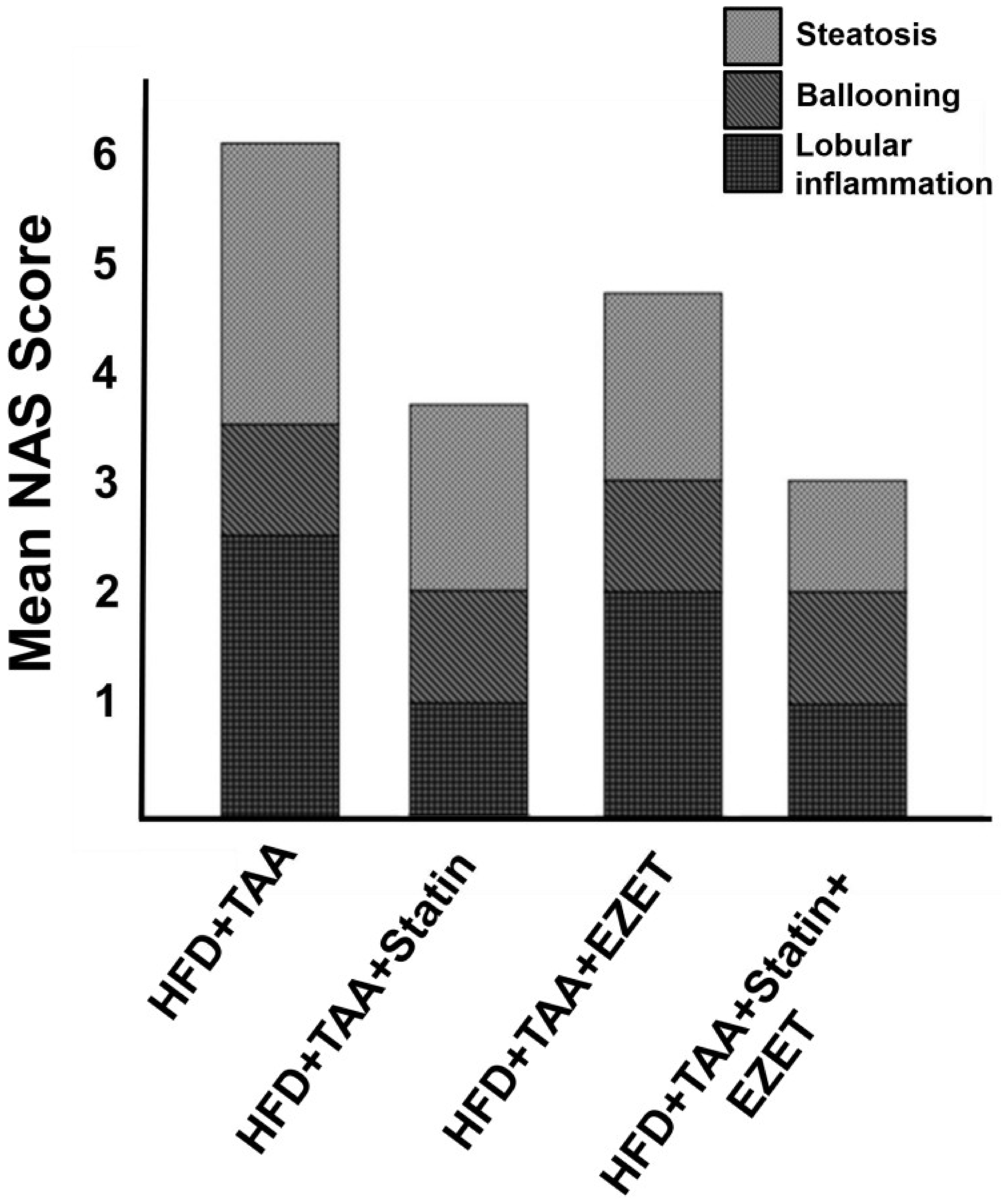


| | Steatosis | Ballooning | Lobular inflammation |
|---|---|---|---|
| **HFD+TAA** | 2.5±0.5 | 1 | 2.5±0.5 |
| **HFD+TAA+Statin** | 1.7±0.47 | 1 | 2 |
| **HFD+TAA+EZET** | 1.7±0.47 | 1 | 1.66 |
| **HFD+TAA+Statin+ EZET** | 1 | 1 | 1 |

**Figure S4. Semi-quantitative histological scoring of hepatic activity using the NASH CRN system.** The NAFLD Activity Score (NAS; sum of steatosis, 0–3; ballooning, 0–2; and lobular inflammation, 0–3) was assessed on H&E-stained sections from three randomly selected animals per group, blinded to group allocation. The stacked bar chart shows mean total NAS, and the table shows mean ± SEM for each component. Fibrosis was assessed separately by Picrosirius red morphometry. Given the small, randomly selected subset, these data are presented descriptively: treatment groups showed a trend toward lower total NAS compared with HFD+TAA, but the differences did not reach statistical significance.

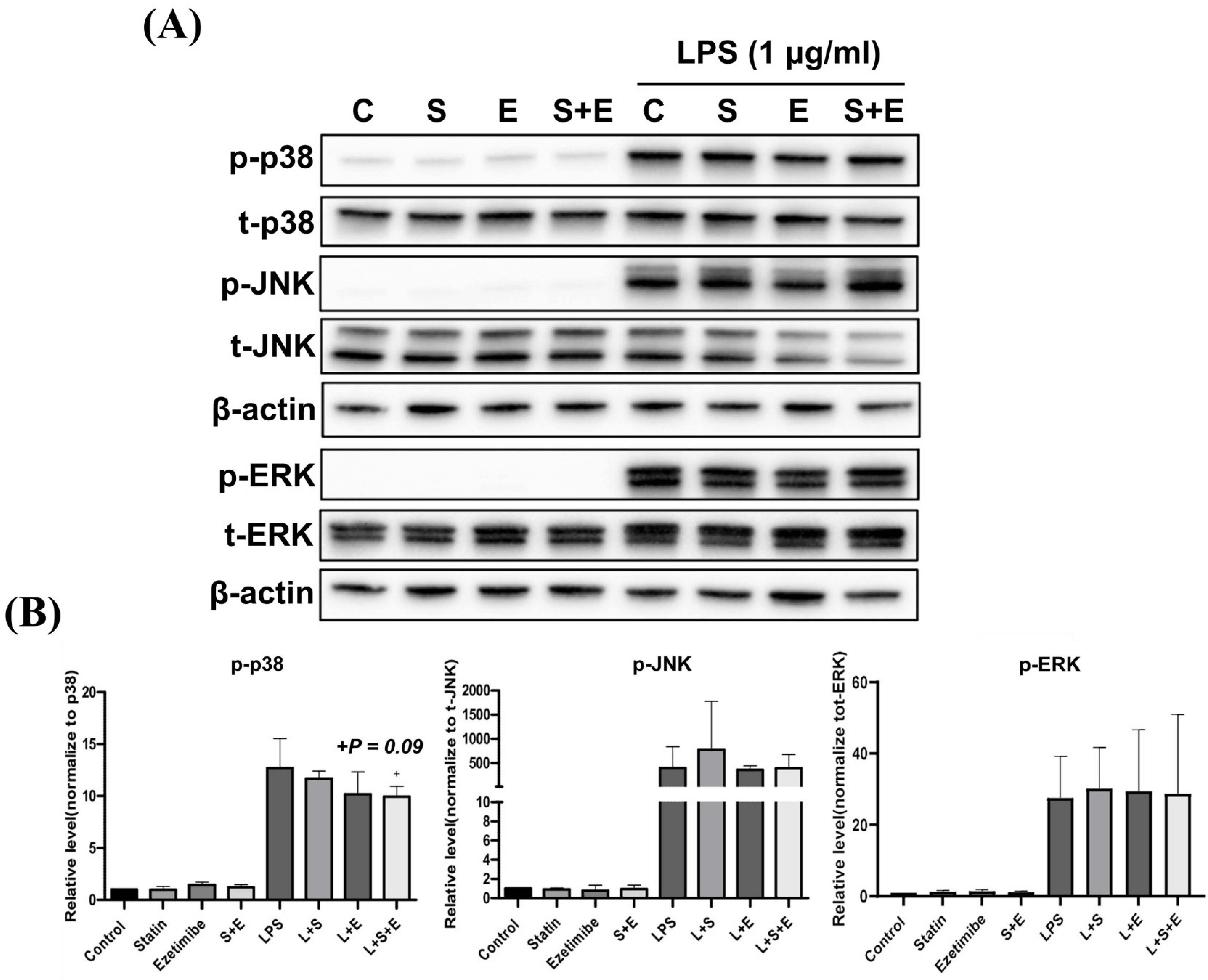


**Figure S5. Statin and ezetimibe combination therapy does not significantly attenuate the MAPK signaling pathway in LPS-stimulated RAW 264.7 macrophages.** Representative Western blot images of phosphorylated (p-) and total (t- or tot-) p38, JNK, and ERK proteins. RAW 264.7 macrophages were treated with simvastatin, ezetimibe, or their combination, either alone (basal condition) or followed by LPS stimulation. Densitometric quantification of p-p38, p-JNK, and p-ERK expression levels, normalized to their respective total protein levels. While LPS stimulation robustly induced the phosphorylation of all three MAPK proteins compared to the basal control, the treatments did not result in a statistically significant reduction of this activation (e.g., $p = 0.09$ for p-p38 in the combination group). These data indicate that the anti-inflammatory mechanism of the treatment is largely independent of the MAPK pathway. Data are presented as the mean ± SEM. Statistical analysis was performed using the Kruskal-Wallis test followed by pairwise Mann-Whitney U tests.

***Abbreviations:*** *S, simvastatin; E, ezetimibe; L or LPS, lipopolysaccharide.*

**Table S1. Specifications of primary and secondary antibodies used in Histomorphology and Immunohistochemical Analysis**

| Primary Antibody | Host | Cat. No. | Company | Dilution |
|---|---|---|---|---|
| Anti-IL-1β | American hamster | sc-12742 | Santa Cruz Biotechnology | 1:1000 |
| Anti-iNOS | Rabbit | NB300-605 | Novus Biologicals | 1:1000 |
| Anti-TNF-α | American hamster | sc-12744 | Santa Cruz Biotechnology | 1:1000 |
| Anti-phospho-p38 MAPK (Thr180/Tyr182) | Rabbit | #4511 | Cell Signaling Technology | 1:1000 |
| Anti-p38 MAPK | Rabbit | #8690 | Cell Signaling Technology | 1:1000 |
| Anti-phospho-SAPK/JNK (Thr183/Tyr185) | Rabbit | #9251 | Cell Signaling Technology | 1:1000 |
| Anti-SAPK/JNK | Rabbit | #9252 | Cell Signaling Technology | 1:1000 |
| Anti-phospho-Erk1/2 (Thr202/Tyr204) | Rabbit | #9101 | Cell Signaling Technology | 1:1000 |
| Anti-Erk1/2 | Rabbit | #9102 | Cell Signaling Technology | 1:1000 |
| Anti-Histone H3 | Rabbit | #9717 | Cell Signaling Technology | 1:1000 |
| Anti-β-actin | Mouse | #58169 | Cell Signaling Technology | 1:1000 |
| Anti-NF-kB | Rabbit | #8242 | Cell Signaling Technology | 1:400 |
| Anti-phospho-NF-kB | Mouse | #13346 | Cell Signaling Technology | 1:1000 |

| Secondary Antibody | Host | Conjugate | Cat. No. | Company |
|---|---|---|---|---|
| Anti-Mouse IgG | Horse | HRP | #7076 | Cell Signaling Technology |
| Anti-Rabbit IgG | Goat | HRP | #7074 | Cell Signaling Technology |
| Anti-American hamster IgG | Mouse | HRP | sc-2789 | Santa Cruz Biotechnology |
| Anti-Rabbit IgG | Donkey | Alexa Fluor 488 | 711-545-152 | Jackson ImmunoResearch |

This table summarizes the detailed information of all primary and secondary antibodies utilized for Western blotting, immunohistochemistry, immunofluorescence, and flow cytometry. The host species, manufacturer, product code, and specific dilution ratios are provided for each antibody to ensure experimental reproducibility.
